# Accessing pluripotent drones through reprogramming of dynamic soft self-healing chemical growth

**DOI:** 10.1093/nsr/nwaf049

**Published:** 2025-02-17

**Authors:** Kecheng Qin, Wei Tang, Xinyu Guo, Huxiu Xu, Yiding Zhong, Yonghao Wang, Qincheng Sheng, Huayong Yang, Jun Zou

**Affiliations:** State Key Laboratory of Fluid Power and Mechatronic Systems, School of Mechanical Engineering, Zhejiang University, Hangzhou 310058, China; State Key Laboratory of Fluid Power and Mechatronic Systems, School of Mechanical Engineering, Zhejiang University, Hangzhou 310058, China; State Key Laboratory of Fluid Power and Mechatronic Systems, School of Mechanical Engineering, Zhejiang University, Hangzhou 310058, China; State Key Laboratory of Fluid Power and Mechatronic Systems, School of Mechanical Engineering, Zhejiang University, Hangzhou 310058, China; State Key Laboratory of Fluid Power and Mechatronic Systems, School of Mechanical Engineering, Zhejiang University, Hangzhou 310058, China; State Key Laboratory of Fluid Power and Mechatronic Systems, School of Mechanical Engineering, Zhejiang University, Hangzhou 310058, China; State Key Laboratory of Fluid Power and Mechatronic Systems, School of Mechanical Engineering, Zhejiang University, Hangzhou 310058, China; State Key Laboratory of Fluid Power and Mechatronic Systems, School of Mechanical Engineering, Zhejiang University, Hangzhou 310058, China; State Key Laboratory of Fluid Power and Mechatronic Systems, School of Mechanical Engineering, Zhejiang University, Hangzhou 310058, China

**Keywords:** pluripotent drones, dynamic reprogramming growth, chemical growth, self-healing, fluidic soft actuation

## Abstract

The functions of drones that are implemented by existing design paradigms are usually fixed and do not have the possibility of further ‘differentiation’. Inspired by the biological concept of pluripotency, here we report a pluripotent drone that can further ‘differentiate’ into a series of drones with different functions to perform a variety of challenging tasks. To realize this concept, we propose a method of reprogrammable dynamic soft self-healing chemical growth (R-growth), by which the pluripotent drone can grow specific ‘organs’ to achieve corresponding functions, and after completing the corresponding tasks, these ‘organs’ can be retracted. Furthermore, these ‘organs’ are able to respond to possible damage through rapid self-healing (∼3.2 s, >1000 times faster than the self-healing of existing similar membranes). R-growth is large-scale (>1.5 m), fast (0.15 m/s), lightweight (∼5 g, 1/20 the weight of traditional micro air pumps), self-contained and free-wheeling. This method can be applied to various existing drones to significantly extend their functions and to enable an unprecedented range of tasks. This work realizes the growth, retraction, and switching of drone ‘organs’ with any function, while such ability of macro robots or humans, to date, only exists in science fiction movies.

## Teaser

Inspired by stem cells, we report a single pluripotent drone that can further ‘develop’ into a series of drones that exhibit different functions to perform a variety of challenging tasks.

## INTRODUCTION

In recent years, the development of drones has been accelerating [1–4], covering daily life, industrial applications, forest protection, field exploration and battlefields. The expansion of their various functions has also attracted much attention as the application scenarios have expanded. Usually, researchers achieve the expansion of drone functions through redundancy [5–9] (adding actuators to the body) and deformation [10–14] (reusing a single actuator). These two strategies are very effective. However, these strategies also fix the functions of the drone, turning it into a drone with a certain specific function. For example, adding a gripper to a drone turns it into a manipulation drone [15]. After installation, these additional mechanisms cannot be retracted or self-modified to achieve other functions, which will cause space redundancy and interfere with flight.

**Figure 1.**
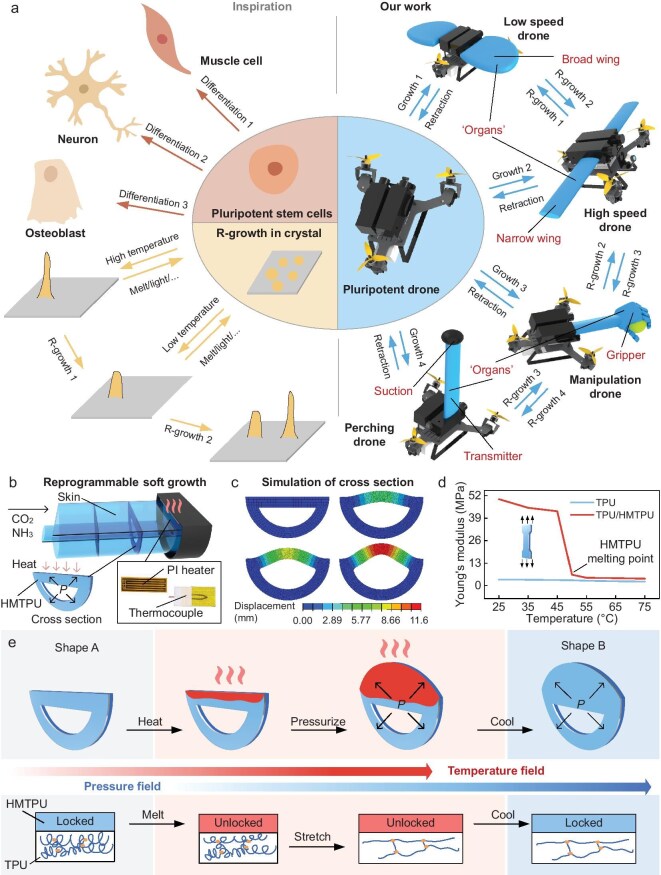
Concept of the pluripotent drone. (a) The design of pluripotent drones is inspired by stem cells and crystal growth. (b) Schematic diagram of the structure for reprogrammable soft growth. The heating unit on the head includes a polyimide heater and a thermocouple, and the upper half of the cross section is heated. (c) Simulation of cross-sectional deformation during reprogrammable growth (Note S4). (d) Uniaxial tensile test of TPU and TPU/HMTPU materials. At 45°C, the Young's modulus of the TPU/HMTPU composite material drops sharply. (e) Schematic diagram of the reprogrammable growth process. The top shows the heating and deformation process of the cross section and the bottom shows the change of the molecular chains.

The basic way of biological development is through growth and differentiation into tissues and organs with various functions. A typical example is that stem cells [16–18] can differentiate into cells with various functions (Fig. 1a). At the same time, the developed organisms have the self-healing ability to cope with various risks. Can this biological pluripotency be introduced into drones so that, when the pluripotent drone needs to perform a certain task or change its movement, it can differentiate to grow ‘organs’ to achieve specific functions? In the microscopic field of materials, changes in inducing factors during crystal growth can lead to changes in the formed crystals, thereby achieving reprogramming growth that is similar to organ differentiation. However, there are currently no such robots in the macro scale that can differentiate and grow ‘organs’ to realize various functions. Such scenarios only exist in fiction stories—that is, showing their pluripotency through growth to achieve various challenging tasks. For example, Nezha in traditional Chinese mythology and Groot in the science-fiction movie *Guardians of the Galaxy* have pluripotency and can grow various organs and retract them when done. They can also self-heal [19,20] quickly and regrow lost arms, enabling them to accomplish tough tasks.

In the field of soft robotics, a class of growing robots [21–30] has attracted widespread attention. However, existing growing methods, including pneumatic and 3D-printing methods, are not suitable for realizing pluripotent drones. Pneumatic growing robots [21–26] can extend continuously along a path, with a circular cross section. The cross-sectional shape is uncontrollable and usually requires a bulky air pump. 3D-printed growing robots [27–30] cannot change their fixed shape after printing, cannot be retracted, grow slowly and have a bulky structure. In addition, they must carry a 3D printer and raw materials, which further increases the weight. Both types of growing robots achieve elongation changes by themselves, which is completely different from our concept of ‘when the pluripotent drone needs to perform a certain task or change its movement, it can differentiate and grow ‘organs’ to achieve specific functions’. Our goal is to find a new method that goes beyond redundancy and deformation to realize pluripotent drones to solve the challenges in existing methods. The growing method used to realize pluripotent drones should have reprogrammable structures, dynamic growing and retracting, and preprogrammable shapes and sizes. Due to the limited load capacity of drones, especially micro drones, the growing method should also be lightweight and cannot be driven by heavy pumps. In addition, as the ‘organs’ are easily damaged when drones work in complex environments, self-healing capabilities should also be available. Therefore, it is extremely challenging to develop a growing method that is suitable for realizing pluripotent drones.

Inspired by pluripotent stem cells and science-fiction movies, we propose the concept of the pluripotent drone that can further ‘differentiate’ into a series of drones that exhibit different functions to perform a variety of challenging tasks. In order to realize this idea, we developed the reprogrammable dynamic soft self-healing chemical growth (R-growth) method, which enables reprogrammable dynamic soft growth induced by pressure and temperature fields, and rapid self-healing through multilayer structures. The pluripotent drone fabricated with the R-growth method can differentiate into a series of drones with different functions in response to different environments or mission requirements. After stem cells differentiate, their functions are usually fixed. One step further than stem cells, pluripotent drones have the ability to retract and switch ‘organs’. At the same time, they can deal with possible damage through self-healing. This single drone has huge advantages, such as in resource-poor or remote areas (in the wild, at sea, in space or on the battlefield), where a single drone can meet evolving and complex needs and applications. Compared with previous methods for realizing the functions of drones (Table S1), our design concept is more advanced. The concept of pluripotent drones can also be extended to the scope of pluripotent robots, providing new possibilities for robot design.

## RESULTS

### Reprogrammable dynamic soft growth

To achieve R-growth, we propose the following three criteria: (i) allowing ‘organs’ to grow anywhere on the drone and allowing reprogramming; (ii) accessible sensitivity to stimuli, so that different ‘organ’ configurations can be obtained online and dynamic adjustment can be achieved; and (iii) the ability to maintain structure after responding to stimuli, dynamically retract ‘organs’ at any time and quickly self-heal after damage.

The differentiation of stem cells is usually not dynamic and cannot be changed once completed. Going one step further than stem cells, the growth, retraction and reconstruction of pluripotent drone ‘organs’ are highly dynamic and can be performed at any time. In order to achieve dynamic reprogrammable growth, multiple cross sections are implanted inside the ‘organ’ to regulate the shape of the ‘organ’ (Fig. S1). Here, a semicircular cross section is used as an example, as shown in Fig. 1b; the ‘organ’ presents a semi-cylindrical shape due to the presence of the semicircular cross section. The cross section is formed by the hot pressing of two layers of films with different properties, one of which is thermoplastic polyurethane (TPU), which is composed of a flexible soft segment and a rigid hard segment. It is less sensitive to temperature, has a smaller Young's modulus and is easy to deform. In contrast, the other layer is hot melting thermoplastic polyurethane (HMTPU), which is more sensitive to temperature, has a larger Young's modulus, is not easy to deform and has a lower melting point. A heating module with proportional-integral-derivative feedback control (Note S2 and Fig. S2) is installed at the front of the ‘organ’ to provide thermal stimulation for reprogrammable growth and can move forward during the growth. The growth of the ‘organ’ is controlled by both temperature and pressure fields (Fig. 1d and e). At the beginning, the head temperature is controlled to 50°C. At this time, the part of HMTPU that is close to the heater melts due to its low melting point and the Young's modulus of this part decreases significantly (Fig. 1d), thereby unlocking the cross-sectional shape. When the temperature field is kept constant, the pressure field begins to take effect. The mixed gas that is produced by the programmable chemical reaction significantly increases the pressure inside the ‘organ’. The cross section is pulled and the randomly bent soft segments of the TPU layer are stretched to deform. Specifically, the part that is close to the heater has a significant drop in the Young's modulus, resulting in a larger deformation, whereas the part that is not heated still maintains the original larger Young's modulus and is not easily deformed. The original semicircular cross section is stretched into a circle. Finally, the heater is turned off while the pressure field is kept constant. As the temperature drops, the HMTPU layer resolidifies to lock in the shape and the shape of the ‘organ’ changes accordingly. As the cross sections almost cover the inside of the ‘organ’ and the head heating module can move forward as the ‘organ’ grows, the proposed reprogramming growth can achieve shape reconstruction at any growth position at any growth stage. The reprogramming of dynamic soft growth turns the drone into a pluripotent drone, which can grow the required ‘organs’ according to the mission and, at the same time, retract these ‘organs’ without affecting its movement. It can also reconstruct the ‘organs’, similarly to Nezha in Chinese mythology.

### Rapid self-healing

**Figure 2.**
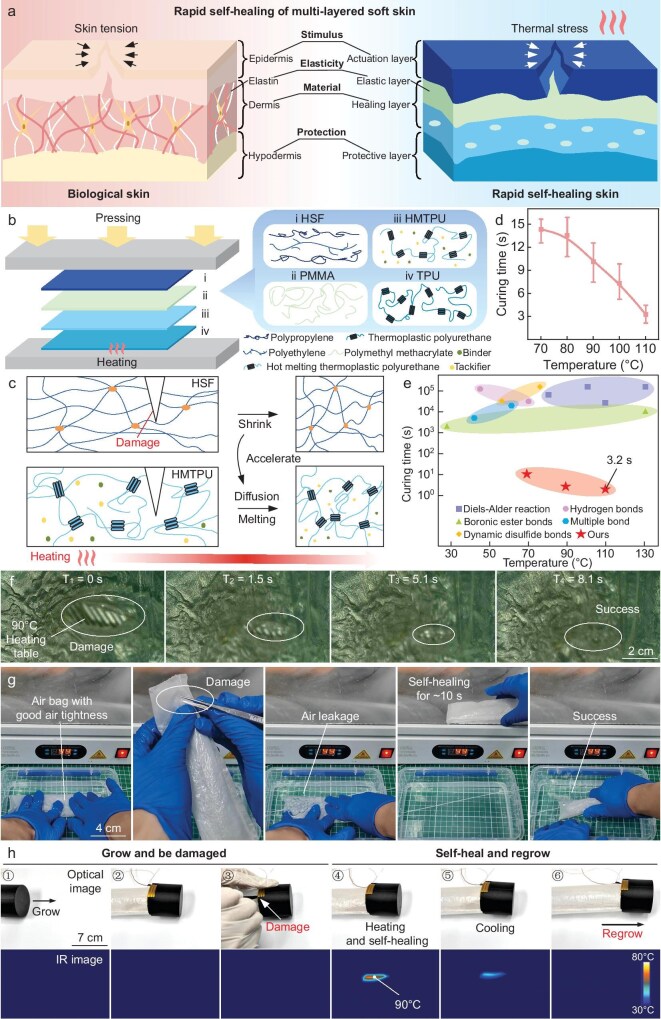
The rapid self-healing skin of the pluripotent drone. (a) Inspired by biological skin, rapid self-healing skin is realized through the synergistic effect of multiple functional films. (b) The manufacturing method of rapid self-healing skin. Rapid self-healing skin can be easily obtained by hot pressing thin film materials with different molecular structures, including HSF, PMMA, HMTPU and TPU. (c) The mechanism of rapid self-healing. The molecular chains of the HSF layer shrink due to the memory effect when heated, which accelerates the diffusion of the molecular chains of the HMTPU layer, thereby accelerating self-healing. (d) Curing time–temperature curve for rapid self-healing skin. (e) Comparison with other self-healing TPUs in curing time (Table S2). (f) Timing diagram of rapid self-healing skin self-healing at 90°C. (g) Timing diagram of self-healing of developed ‘organ’ based on rapid self-healing skin. Developed ‘organ’ leaks after being damaged and does not leak after rapid self-healing. (h) The timing diagram of the self-healing of the growing ‘organ’. After being damaged, the ‘organ’ quickly self-heals and continues to grow.

The ‘organs’ that are grown by pluripotent drones are at risk of damage and self-healing after damage remains a challenge. Traditional TPU self-healing is mainly based on its physical properties—especially its block copolymer structure that is composed of hard segments and soft segments. The soft segment is fluid when heated, which helps the self-healing process. In recent years, self-healing TPU has gradually emerged. These elastomers achieve self-healing through reversible non-covalent bonds (such as π–π stacking, hydrogen bonds, ionic interactions and coordination bonds) [31–33] or reversible covalent bonds (such as disulfide bonds, borate bonds, acetylhydrazone bonds and Diels–Alder dynamic covalent bonds) [34–36]. However, both traditional and self-healing TPU need hours to self-heal. The fluidity after heating is the biggest factor limiting the speed of self-healing. The self-healing ability of biological skin is remarkable. After wounded, the multilayers of the skin play different roles [37] and they work together to accelerate wound healing (Fig. 2a). Specifically, the epidermis is the outermost layer of the skin. During the wound-healing process, external pressure stimulates the epidermis to promote coagulation and vasoconstriction. Epidermal cells migrate rapidly and cover the wound surface to form a protective layer. The dermis contains a large number of fibroblasts, collagen and elastin. These components provide strength and elasticity to the skin and also provide cell resources for wound healing. The hypodermis plays a protective role when the skin is damaged, reducing external damage to deep tissues. Inspired by biological skin, we propose a rapid self-healing skin that consists of multiple layers of functional films and is the main building material for the ‘organs’. The outermost layer is the actuation layer, which can sense external stimuli similarly to the epidermis and can quickly stack materials at the wound site. The elastic layer, like elastin, provides skin tension for the rapid self-healing skin, the healing layer provides healing resources similarly to the dermis and the protective layer provides deep protection similarly to the hypodermis.

The rapid self-healing skin of our pluripotent drone is fabricated by the heat pressing of multiple layers of films (Fig. 2b and Note S1), which is a readily accessible fabrication method. The actuation layer is a hot shrink film (HSF) with shape memory effect. It is co-extruded with polyethylene (PE) as the middle layer and polypropylene (PP) as the inner and outer layers. During the production process of HSF, the polymer film is heated to above its glass transition temperature or melting point and then rapidly stretched. This causes the molecular chains to be oriented along the stretching direction. The stretched film is rapidly cooled to fix the oriented molecular chain structure. After thermal stimulation, the stretched amorphous chains are released by melting and return to their original shape. The elastic layer is a polymethyl methacrylate (PMMA) film, which has a certain degree of flexibility within its molecular structure. Its polymer chains can be stretched and deformed to a certain extent when subjected to force without breaking easily and can return to their original shape when the external force is removed. Its surface has adhesive to bond the actuation layer and the healing layer. The healing layer is HMTPU, which has a low melting point. Driven by thermal motion, the HMTPU molecular chains in the damaged area will undergo wetting, diffusion and rearrangement, achieving surface healing through physical contact and intermolecular interactions (such as van der Waals forces). The protective layer is TPU, which has a high melting point and will not melt due to thermal stimulation. At the same time, it has good compatibility with the healing layer. After an injury, we apply thermal stimulation to the wound, the HMTPU in the healing layer will melt quickly, the physical cross links between the molecular chains will be destroyed and it will diffuse into the wound. At the same time, the HSF of the actuation layer is quickly released under thermal stimulation and shrinks toward the wound. As the layers are tightly bonded to each other, the thermal contraction of the actuation layer will also drive the contraction of the healing layer, significantly accelerating the diffusion of the molecular chains (Fig. 2c) and thereby greatly shortening the self-healing time. The self-healing time of wounds (3 mm × 3 mm) is tested at different temperatures and the fastest can take only 3.2 s (Fig. 2d), which is >1000 times faster than the self-healing of the same type of materials (Fig. 2e and Table S2). In order to verify the effectiveness of the rapid self-healing, we conduct damage and self-healing tests on the rapid self-healing skin film (Fig. 2f), the developed ‘organ’ (Fig. 2g and Movie S1) and the growing ‘organ’ (Fig. 2h and Movie S2), and they all demonstrate rapid self-healing capabilities. The heating device is installed on the growing ‘organ’ and can move with the growth and retraction of the growing ‘organ’ to heal the entire growing ‘organ’. The rapid self-healing skin enables the pluripotent drone to have biological-like self-healing capabilities to cope with various risks during the execution of tasks, such as being punctured by sharp objects (Fig. S15 and Movie S12). In actual use, in order to cope with more severe and complex environments, we can attach a more stable protective layer (e.g. nylon cloth, silicone, etc.) to the outer layer of these materials.

### Programmable chemical growth mechanism

**Figure 3.**
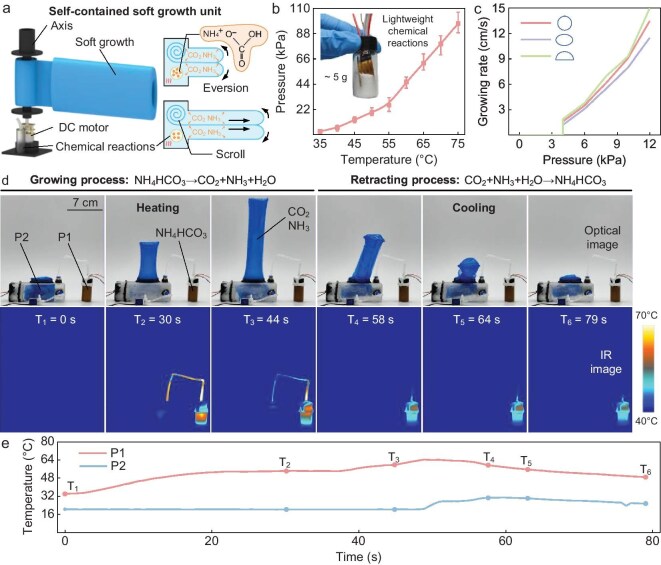
Chemical growth mechanism. (a) Chemical growth mechanism and self-contained soft growth unit based on this mechanism. (b) Thermal decomposition pressure–temperature curve of ammonium bicarbonate. The weight of the ammonium bicarbonate solid is 5 g. (c) Growth rate–pressure curves of ‘organs’ with different cross-sectional shapes. (d and e) The remote-actuation ability of the chemical growth mechanism, the timing diagram (including optical image and infrared imaging image) and the temperature curve of the chemical growth mechanism that is driving the growth and retraction of the ‘organ’. P_1_ is in the external ammonium bicarbonate reaction vessel and P_2_ is in the growth unit. The temperature of P_2_ is significantly lower than the temperature of P_1_.

We construct a programmable chemical growth mechanism that contains ammonium salts, and manufacture a lightweight, self-contained growth unit that is based on this mechanism (Fig. 3a and Note S1). The growth unit is built with a large amount of ammonium bicarbonate powder, the decomposition of which is a reversible reaction that can be controlled by temperature. When the temperature rises, ammonium bicarbonate decomposes into carbon dioxide, ammonia and water. According to the ideal gas equation, the pressure in the enclosed space rises rapidly, providing power for ‘organ’ growth. When the temperature drops, the chemical gas resynthesizes into ammonium bicarbonate powder and the pressure of the system drops sharply, creating conditions for the recovery of the ‘organs’. When the system temperature is set to 75°C, the thermal decomposition of only 5 g of ammonium bicarbonate powder can provide a pressure of >100 kPa (Fig. 3b), which far exceeds the pressure required for the growth of ‘organs’ of different cross sections (∼ 4 kPa, Fig. 3c). With the help of the ammonium bicarbonate chemical growth mechanism, the growth rate can reach ≤15 cm/s, which is sufficient to meet the needs of drones. We establish a theoretical model of the chemical growth mechanism to guide the application development of the chemical growth mechanism (Note S7).

Considering that the ‘organ’ is formed by the hot pressing of multilayer films, the temperature that it can withstand should not be >120°C. Therefore, during the heating of the chemical reaction, the temperature at the ‘organ’ should not be too high. The chemical growth mechanism is driven by the thermal decomposition of ammonium bicarbonate to produce gas, thereby increasing the pressure of the system, and can be remotely driven. To better observe the remote-actuation performance of the chemical growth mechanism, ammonium bicarbonate powder is externalized and connected to the ‘organ’ system by using a catheter (Fig. 3d and Movie S3). During the growth process, the ammonium bicarbonate is heated and the maximum temperature can reach 70°C, while the temperature inside the ‘organ’ is only slightly higher than room temperature (∼40°C). When the temperature drops, the gas is converted back into a solid, the internal scroll is recovered and the ‘organ’ retracts.

**Figure 4.**
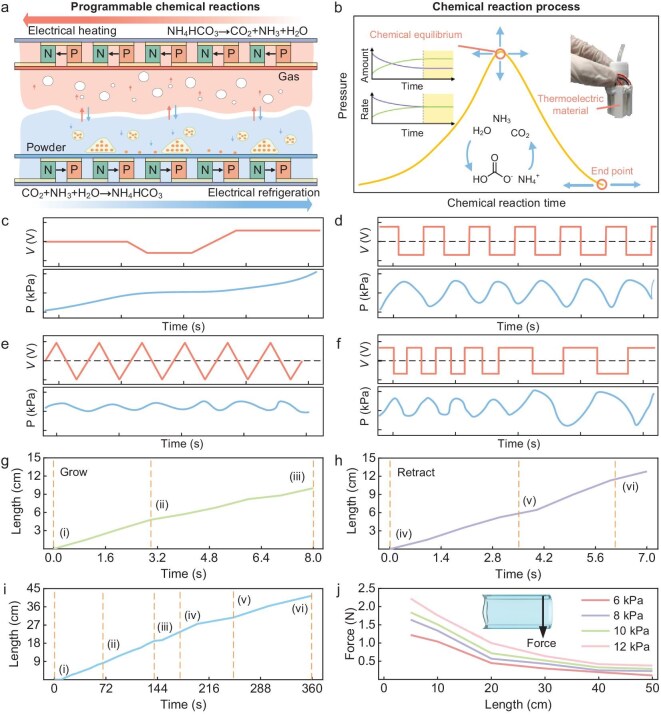
Programmable chemical growth. (a) The principle of programmable chemical growth, which mainly includes thermoelectric materials that can cool or heat, and the reversible decomposition reaction of the ammonium bicarbonate. When the thermoelectric material heats, the temperature rises and the mixed gas is generated. When the thermoelectric material cools, the temperature drops and solid ammonium bicarbonate is generated. (b) Regulation of chemical reaction equilibrium. In the reversible reaction of ammonium bicarbonate, the change in reaction conditions will realize the full-directional regulation of chemical equilibrium. (c–f) The programming process of programmable chemical growth. By programming the voltage, the pressure curve is programmed. Different voltage curves correspond to different pressure curves. (g, h) Growth and retraction test of the self-contained growth unit. (i) Underwater test of the self-contained growth unit. (j) Rigidity test of the ‘organ’, with a load added at the end.

When the initial conditions are constant, the process of chemical reactions is fixed and cannot meet the requirements for high controllability of the driving source for R-growth. We propose a programmable chemical drive (Fig. 4a) by using thermoelectric materials to perform thermal management on reversible chemical reactions, manipulate the movement of chemical equilibrium (Fig. 4b) and thus regulate the driving pressure. Thermoelectric materials are based on the Peltier effect [38]. When current passes through a contact point that is composed of two different metals, electrons jump from one material to another, absorbing or releasing energy according to the different energy levels in the two materials. If the electron releases energy, then the contact point will absorb heat; if the electron absorbs energy, then the contact point will release heat. A change in the direction of the current will change the behavior of absorbing or releasing heat, and a change in the magnitude of the current will change the heating or cooling power of the thermoelectric material. When the current flows from the P-type semiconductor to the N-type semiconductor, the thermoelectric material generates heat, ammonium bicarbonate is decomposed into a mixed gas and the system pressure increases; when the current flows from the N-type semiconductor to the P-type semiconductor, the thermoelectric material cools, the mixed gas is resynthesized into ammonium bicarbonate and the system pressure decreases. When the thermal decomposition of ammonium bicarbonate reaches chemical equilibrium, the increase or decrease in the current can drive the chemical equilibrium point and the end point to move in all directions, thereby changing the amount of the product and obtaining the driving pressure required for growth. The direction, magnitude and rate of change of the current will affect the heating or cooling power of the thermoelectric material, thereby affecting the progress of the chemical reaction and the final driving pressure. Therefore, we can program the voltage signal. Under different voltage waveforms, the chemical equilibrium is manipulated and ultimately a pressure curve with different characteristics can be obtained (Fig. 4c). This is programmable chemical drive.

The integrated self-contained growth unit is tested, with a growth rate (limited by the motor) of 1.25 cm/s and a retraction rate of 1.83 cm/s (Fig. 4g and h, Note S6 and Fig. S5). Traditional rigid air pumps cannot be used underwater but the self-contained unit is still applicable underwater (Fig. 4i and Fig. S6). During the flight of the drone, considering that the ‘organ’ that extends outward will be subject to wind resistance and load, we test the end load capacity of the ‘organ’ (Fig. 4j). The end load capacity increases with increasing pressure and decreases with increasing length. A circular cross-section ‘organ’ with a diameter of 3.5 cm can withstand an end load of >2 N at a pressure of 12 kPa. Compared with traditional rigid air pumps, the self-contained growth unit that is designed based on the chemical growth mechanism has a significant reduction in weight and volume (1/20 the weight of traditional micro air pumps) and is applicable in complex environments such as swamps, underwater and mud. Compared with physical actuation that is based on a gas–liquid phase change [39,40], the chemical growth mechanism has more advantages in remote actuation. Typical gas–liquid phase-change materials, such as fluorinated liquids, will liquefy rapidly at lower temperatures at the far end and cannot be remotely driven. Compared with traditional chemical reactions [41,42], programmable chemical reactions are highly controllable and meet the growth needs of pluripotent drones.

### Customizable preprogrammed shapes

**Figure 5.**
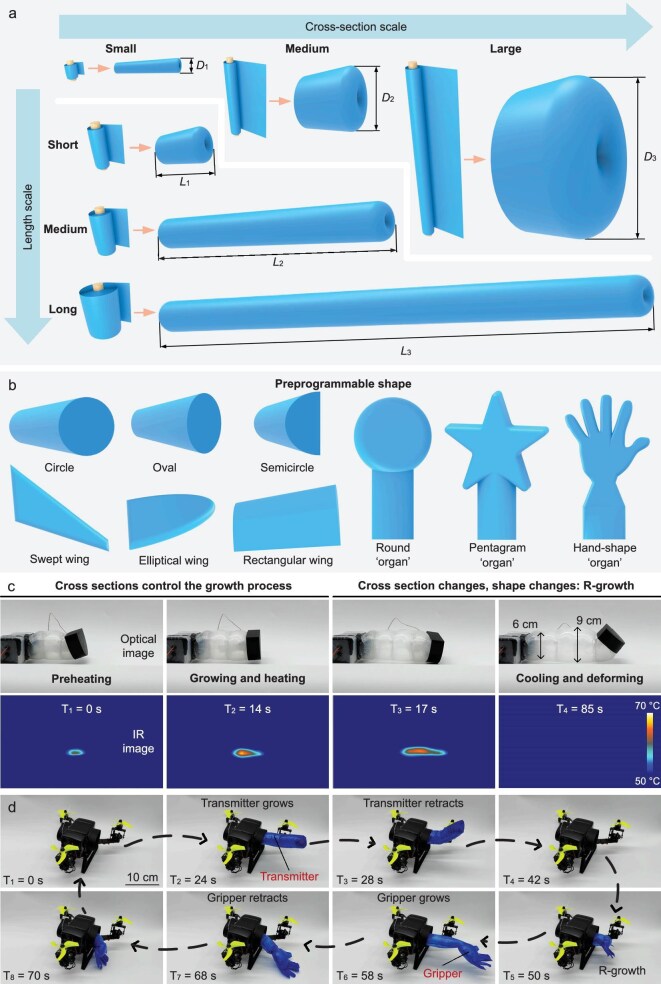
Customizable preprogramming shapes of the ‘organ’. (a) Preprogramming sizes. By adjusting the size of the original scroll, the size of the ‘organ’ can be preprogrammed. (b) Preprogramming shapes. By adjusting the cross-sectional shape and outer contour, the shapes of the ‘organ’ can be preprogrammed, including wing-shaped, spherical, hand-shaped ‘organs’, etc. (c) Reprogramming adjustment. Under the combined action of pressure field and temperature field, the diameter of the cylindrical ‘organ’ increases from 6 to 9 cm. (d) Reprogramming switch. The pluripotent drone can completely retract the developed ‘organ’ and grow a hand-shaped ‘organ’.

The ‘organs’ can be customized and preprogrammed into various sizes and shapes to achieve the diverse functional ‘differentiation’ of pluripotent drones of different scales. The preprogrammed ‘organ’ size depends on the customization of the scroll size (Fig. 5a). The cross-sectional size of the ‘organ’ increases with an increase in the scroll height and the length of the ‘organ’ increases as the cross-sectional area of the scroll increases. The preprogrammable ‘organ’ size can meet the functional differentiation needs of drones of different scales. The preprogramming of shapes has also attracted much attention for achieving the multifunctional differentiation of pluripotent drones. The preprogramming of shapes can be subdivided into the preprogramming of cross sections and the preprogramming of contours (Fig. 5b). The cross section can be customized into various shapes such as elliptical, circular or semicircular according to needs. When the ‘organs’ are retracted, their cross sections will naturally stack so that, regardless of the original shape, they will eventually present a unified appearance. In addition, according to the specific differentiation needs of the drone, the cross-sectional design can be further expanded to accommodate more diverse forms. The design of the wing is crucial to the performance of drones because it directly affects the aerodynamic characteristics of drones, including lift, drag, stability and maneuverability. By preprogramming the contours of the ‘organs’ into having different wing shapes, pluripotent drones can grow and differentiate different wing shapes to adapt to different aerodynamic environments or switch between different wing shapes. Specifically, when the drone is flying at low speed, it can grow rectangular wings to improve the operability of the drone; when the drone needs to fly at high speed, it can be reprogrammed to grow swept wings to reduce wingtip vortices and improve flight efficiency; when the drone needs to fly long distances, it can be reprogrammed to grow elliptical wings to reduce induced drag. In addition to improving flight capabilities, the preprogramming of the ‘organ’ contour can also be used for drone operations, such as hand shapes for grasping, circles for sensing the external environment or special shapes (Fig. S7 and Movie 4) such as a pentagram ‘organ’ for performing special tasks. It should be noted that the realization of the final function does not rely on the specific design of a single element, but is achieved through the coordinated optimization of the size, cross section, and contour of the ‘organ’. The preprogrammed ‘organ’ is made of thin film, which is very lightweight and suitable for drones with limited loads. Based on preprogrammed ‘organs’, drones can grow functional ‘organs’ that are adapted to mission requirements, and can adjust and switch ‘organs’ through reprogramming growth (including reprogramming adjustment and reprogramming switching) to adapt to changes in demand.

Reprogramming growth can be divided into small-scale adjustment and large-scale switching. As shown in Fig. 1e, reprogramming adjustment achieves changes in ‘organs’ by manipulating cross sections. Specifically, under the dual effects of temperature field and pressure field, the circular cross section is reprogrammed to grow from a height of 6 cm to a height of 9 cm (Fig. 5c, Fig. S3, Movie S5 and Note S3). When the drone needs to switch functions on a large scale, reprogramming adjustment can no longer meet the needs. We propose a reprogramming switching method, which is to retract the original ‘organ’ and grow an ‘organ’ with new function. For example, a drone can grow a cylindrical actuator. When it needs to switch to a grasping function, the original cylindrical actuator will be completely retracted and a new gripper will grow (Fig. 5d, Fig. S4, Movie S6 and Note S5). The preprogramming and reprogramming growth of ‘organs’ can achieve real-time regulation of specific ‘organ’ shapes, providing an ideal solution for pluripotent drones to differentiate into specific functions and switch functions.

### Pluripotent drones

Through R-growth, we have made various types of drones into pluripotent drones, including fixed-wing, multirotor and vertical take-off and landing (VTOL) drones. They can grow and retract various ‘organs’, including wings, grippers, signal transmitters, etc., and can also switch between these ‘organs’ through R-growth. Pluripotent drones have a wide range of potential capabilities, including locomotion, multi-environment and manipulation capabilities.

**Figure 6.**
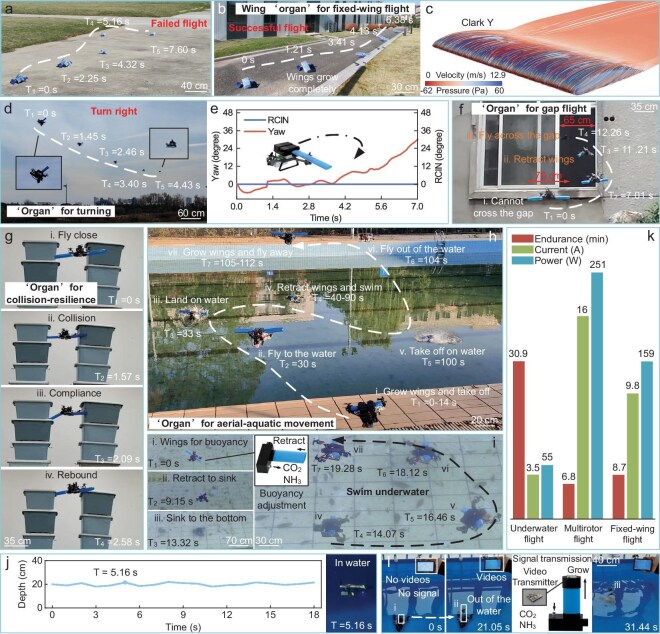
Locomotion and multi-environment capability of the pluripotent drone. (a) Without the growth of a wing shape, the pluripotent drone fails to take off. (b) The pluripotent drone grows wings and takes off successfully. (c) Aerodynamic simulation results of the wing-shaped ‘organ’. The wing shape is Clark-Y and the scale is speed (m/s) and static pressure (Pa). (d, e) The pluripotent drone retracts the ‘organ’ and completes a 50° turn. (f) The pluripotent drone retracts the ‘organ’ and flies across a narrow gap. Without retracting the ‘organ’, the width of the unmanned aerial vehicle is 70 cm, which is larger than the width of the gap (65 cm). (g) The pluripotent drone grows a soft ‘organ’ to be collision-resilient. (h) The pluripotent drone grows an ‘organ’ and crosses the air–water interface. (i) The pluripotent drone grows an ‘organ’ to adjust buoyancy and swims underwater. (j) Depth-fixed swimming of a pluripotent drone (Fig. S13). (k) Energy consumption of a pluripotent drone in different modes. (l) A pluripotent drone grows ‘organs’ to transmit underwater video signals.

A 51-g lightweight fixed-wing pluripotent drone uses a 5-g programmable chemical drive unit (Fig. S8). The drone can only move on the ground without wings but, once it grows wings, it can take off and glide in the air (Fig. 6a and b, Fig. S9 and Movie S7). Simulation tests show that the grown wings can provide sufficient lift (Fig. 6c) and the drone can achieve flexible steering through the dynamic growth and retraction of the wings, and even perform 50° yaw control during flight (Fig. 6d and e). In addition, the drone also has reverse differentiation capabilities and can detect through narrow spaces (Fig. 6f and Movie S8). In the event of a collision, the soft ‘organ’ of the drone can provide protection and the built-in barometer can sense obstacles and enhance obstacle-avoidance capabilities (Fig. 6g).

The pluripotent drone can grow different ‘organs’ according to environmental needs to improve adaptability. In the air, the drone will grow wings to provide lift; underwater, it will retract the wings to reduce resistance (Fig. 6h and Movie S9) and can grow buoyancy adjustment modules (Fig. 6i) to adapt to different water depths and achieve stable swimming (Fig. 6j). The drone can also perform repeatable conversions between water and air, maintaining high energy efficiency in each mode (Fig. 6k). In addition, the drone can also grow image transmission probes to solve the problem of underwater signal transmission and realize real-time transmission of video signals below the water surface (Fig. 6l).

**Figure 7.**
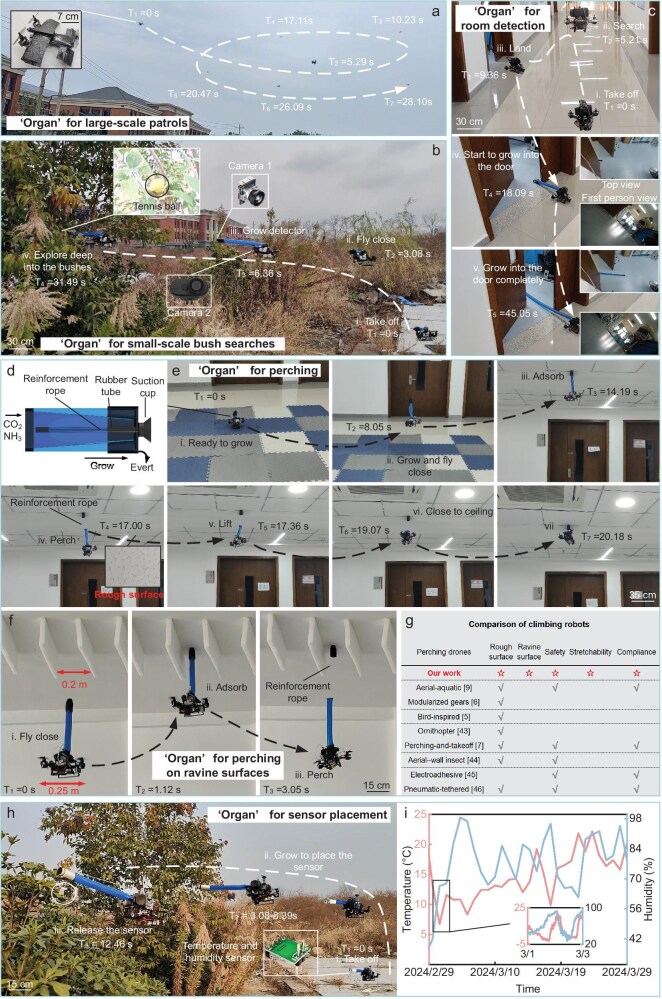
Manipulation capability of the pluripotent drone. (a, b) The pluripotent drone grows ‘organs’ for detection, including large-scale patrols and small-scale bush searches. (c) The pluripotent drone grows large-sized ‘organs’ (1.5 m) for room detection. (d) Schematic diagram of the structure of the adsorptive ‘organ’; the pump provides negative pressure to the suction cup through a silicone tube. (e) The pluripotent drone grows adsorptive ‘organs’ for perching. (f) The pluripotent drone growing adsorptive ‘organs’ and perching on the ravine surface. (g) Comparison with other perching drones [5–7,9,43–46]. (h, i) The pluripotent drone grows ‘organs’ deep into the bushes for sensor placement and completes a month of temperature and humidity monitoring.

Pluripotent drones enhance their manipulation capabilities by growing ‘organs’. The R-growth capability of the drone improves its efficiency in large-scale patrol exploration (Fig. 7a) and enables it to carry cameras and conduct exploration in complex environments such as bushes (Fig. 7b and Note S13). When performing corridor patrol missions, the drone can reprogram its ‘organ’ on a large scale to grow to 1.5 m in length (Fig. 7c and Movie S10) and enter the room through the door gap for observation and rapid response. The drone can also grow suction cups on the ceiling to achieve stable perching (Fig. 7e, Movie S11 and Note S14) and can even adhere to the surface with deep ravines, demonstrating excellent safety and adaptability (Fig. 7f and g). Compared with rigid perching structures [5–7,9,43–46], perching through ‘R-growth’ is soft and has compliance. The drone does not need precise positioning to perch, so it is safer. Compared with flexible suction-cup perching structures, perching through ‘R-growth’ can be flexibly extended and retracted, and the distance between the drone and the wall can be adjusted according to different tasks, so the drone can perch on ravine surfaces. In addition, in the jungle sensor placement task, the drone can keep the body away from danger and grow actuators with sensors to penetrate into complex environments for monitoring (Fig. 7h and i).

## DISCUSSION

**Figure 8.**
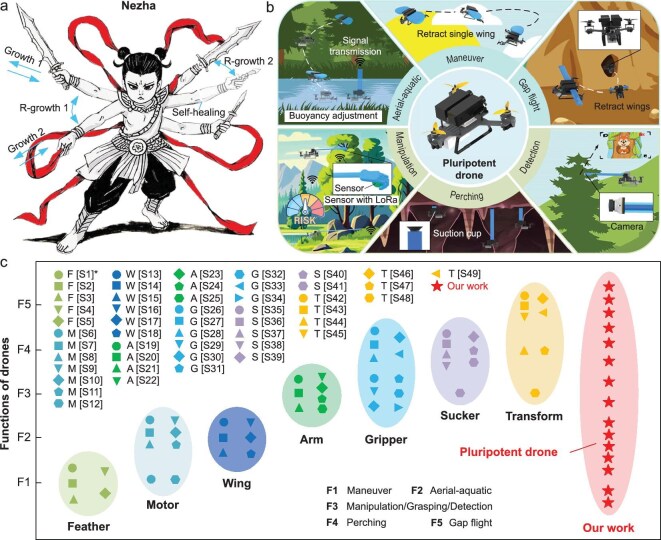
Growth scenes in myths and comparison with other drones. (a) The growth ability of Nezha in Chinese mythology. Nezha can grow different arms, switch between them at will and also has self-healing capabilities. (b) The application of a pluripotent drone. The pluripotent drone can grow ‘organs’ to perform different tasks, including aerial–aquatic locomotion, maneuver, gap flight, manipulation, perching and detection, and switch between them at will. (c) Comparison with other drones (Table S1). Other drones can only complete the existing limited functions, whereas our pluripotent drone can achieve the growth, disappearance and reconstruction of functions. *: S[XX] refers to reference S[XX] in the Supplementary file.

Our work enables some growth scenes that are seen in science-fiction movies and myths to become a reality, achieving effects similar to those of Nezha (Fig. 8a) in Chinese mythology. Through reprogrammable dynamic soft growth induced by pressure and temperature fields, rapid self-healing achieved through multilayer structures, programmable chemical growth mechanism and preprogrammed shape and size, we have realized a pluripotent drone. Unlike previous robot design paradigms, the structure and function of our pluripotent drone are not fixed, and the pluripotent drone can achieve on-demand growth, retraction and switching of ‘organs’. Thus, the pluripotent drone can achieve various functions and be applied to various scenarios (Fig. 8b). Compared with previous methods for realizing the drone functions (Fig. 8c and Table S1), our design concept is more advanced, not only encompassing their existing functions, but also realizing the removal and reconstruction of functions. The pluripotent drone design concept can also be extended to other robot systems, providing a new direction for robot design.

## MATERIALS AND METHODS

Detailed methods are given in the online Supplementary materials.

## Supplementary Material

nwaf049_Supplemental_Files

## Data Availability

All data needed to evaluate the conclusions in the paper are present in the paper and/or the Supplementary materials.
