# Supplementary material for: Accessing pluripotent drones through reprogramming of dynamic soft self-healing chemical growth
Source: Natl Sci Rev. 2025 Feb 17;12(6):nwaf049. doi: 10.1093/nsr/nwaf049 (PMC12051850; doi:10.1093/nsr/nwaf049)
Supplement: nwaf049_Supplemental_Files [file nwaf049_supplemental_files.zip › Supplementary Materials 0212.pdf]

Supplementary Materials

Accessing pluripotent drones through reprogramming of dynamic soft self-healing chemical growth

Kecheng Qin et al.

Correspondence to: Wei Tang: weitang@zju.edu.cn; Jun Zou: junzou@zju.edu.cn

This PDF file includes:

Supplementary Notes
Fig. S1. A completed wing's image.
Fig. S2. A temperature regulation system employing Proportional-Integral-Derivative (PID) algorithm.
Fig. S3. Shape alteration in response to varying pressure through reprogramming growth.
Fig. S4. Principles of reprogramming switching. The growth unit contains multiple scrolls that can grow into "organs", which are pre-programmed into different shapes.
Fig. S5. Growth velocity tests under varying pressures.
Fig. S6. Images of the "organ" growing underwater.
Fig. S7. Growth images of "organs" with pre-programmed shapes.
Fig. S8. Architecture of a lightweight pluripotent fixed-wing drone.
Fig. S9. Indoor testing scenario for the fixed-wing drone's performance.
Fig. S10. Architecture of a tri-rotor drone.
Fig. S11. Waterproof drone hardware.
Fig. S12. Thrust measurement experiment for a brushless motor equipped with a 3-inch propeller.
Fig. S13. Depth-fixed swimming images of the drone.
Fig. S14. Architecture of a quadrotor drone.
Fig. S15. "organ" self-healing of a quadrotor drone.
Table S1. Comparison with existing multi-functional drones.
Table S2. Comparison with existing TPU self-healing methods.
Legends for Movies S1 to S12
Supplementary references [*S1-S61*]

Other Supplementary Materials for this manuscript include the following:

Movie S1. Self-healing of a developed "organ".
Movie S2. Self-healing of a growing "organ".
Movie S3. Chemical growth mechanism.
Movie S4. Customizable preprogramming shapes of the "organ".
Movie S5. Reprogramming adjustment of the "organ".
Movie S6. Reprogramming switch of the "organ".

Movie S7. Pluripotent drone growing “organs” - wings.
Movie S8. Pluripotent drone retracting “organs” for gap flight.
Movie S9. Pluripotent drone growing “organs” for aerial-aquatic movement.
Movie S10. Pluripotent drone growing a large-scale “organ” for room detection.
Movie S11. Pluripotent drone growing an adsorptive “organ” for perching.
Movie S12. Pluripotent drone self-healing the damaged “organ”.

Supplementary Notes

1. Rapid self-healing skin and growth unit

The actuation layer is 0.02 mm thick HSF (Zhongxin, China), the elastic layer is 0.25 mm thick PMMA film (Mingshen, China), the healing layer is 0.03 mm thick HMTPU film (Xingxia, China), and the protective layer is 0.05 mm thick TPU film (Tepuyou, China). In order to achieve uniform growth and retraction of the "organ", we use a DC motor to limit the growth and contraction speed. The working voltage is 12 V and has a stable rotation speed of 30 revolutions per minute. Ammonium bicarbonate solid needs to be pulverized before chemical reaction to form fine powder, which can significantly increase its contact area with reactants, thereby improving reaction efficiency. Thermoelectric materials (TECooler Technology, China) are used for heating and cooling. K-type thermocouples (Kaipusen, China) are used to detect the temperature within the growth unit. The structural connectors of the growth unit are made of polylactic acid. (Bambu Lab, China). A PI film (width, 10 mm; Rebainian, China) is used as a heat source for self-healing and reprogramming adjustments.

2. PID control in reprogramming growth

The temperature control system adopts the PID control strategy, as shown in fig. S2. This system has closed-loop feedback that can respond quickly and accurately adjust the temperature. The system generates control instructions using the embedded PID algorithm by detecting and comparing the difference between the set temperature and the actual measured value, thereby manipulating the switching state of the solid-state relay and the heating process. The mathematical model of PID control can be expressed as:

$$u(t) = K_p e(t) + K_i \int_0^t e(\tau)d\tau + K_d \frac{de(t)}{dt} \tag{1}$$

Where K_p, $K_{i,}$ and K_d represent the proportional gain, integral gain, and differential gain respectively, e(t) represents the control deviation (i.e. the difference between the set temperature *r(t)* and the actual measured temperature *y(t)*), and *u(t)* is the output control signal.

3. Reprogramming adjustment

During reprogramming adjustment, the cross section is under the control of the temperature field and pressure field. Specifically, the temperature field dominates the phase transformation process of HMTPU, thereby achieving changes in the cross-section Young's modulus. The pressure field is responsible for deforming the cross section. We test the height of the section under various pressure conditions after the temperature field took effect (fig. S3), and find that as the pressure gradually increases, deformation becomes more significant. The temperature field of the head adopts a PID control algorithm, using a PI heater for heating and a K-type thermocouple for temperature feedback (fig. S2). The pressure field mainly uses thermoelectric materials to thermally manage the reversible chemical reaction of ammonium bicarbonate to achieve different pressures. The thermal management of the thermoelectric material adopts a bidirectional PID control algorithm. We add an H-bridge circuit to the thermoelectric material to provide positive and negative voltages to control whether the thermoelectric material is heating or cooling, and a K-type thermocouple is used for temperature feedback.

4. Programming growth simulation

ABAQUS is used to simulate the cross-sectional deformation of the “organ” (Fig. 1c). During the simulation, the Young's modulus (stretching rate less than 20%) of the material decreases in the upper region affected by heat. In order to obtain the mechanical behavior of the material, we test the uniaxial tensile properties of TPU/HMTPU material at different ambient temperatures on a universal testing machine (DGD-50, China). The dimensions of these test

specimens strictly follow the 643 class A specifications in the ASTM D412 standard. Using these experimental results, we construct an elastic model of the material in ABAQUS and fit the elastic moduli of the upper and lower layers, respectively, and the values obtained are 5 MPa and 50 MPa, respectively, which are shown in Fig. 1d.

5. Reprogramming switch

Reprogramming adjustment means that the pluripotent drone completely retracts the original "organs" and grows new "organs". We provide a schematic diagram of reprogramming adjustment (fig. S4). Multiple reprogrammed scrolls are placed inside the chemical growth unit. The programmable chemical growth mechanism cooperates with the motor to complete the reprogramming adjustment.

6. Growth test

We build a test platform (fig. S5) to evaluate the growth rate of "organs" under different pressure conditions. The test platform consists of a ruler and a barometer (ZSE30AF-01-P-L, Qingwen, China) that accurately controls the pressure. The barometer can achieve a pressure control accuracy of up to 0.1 kPa. For each measurement, we perform five sets of experiments and calculate their average values to ensure the accuracy and reliability of the results. The pressure tests involved in the article are all completed by a pressure gauge (AS511B, Xima, China). All thermal imaging experiments were completed by a thermal imager (A615, FLIR, USA).

7. Mathematics model of chemical growth mechanism

Ideal gas equation，

$$PV = nRT \tag{2}$$

The pressure P can be generated for pneumatics by regulating several factors, such as the gas volume V, the number of moles of gas n, the molar gas constant R, and the temperature T, individually or in combination. The reversible chemical reaction of ammonium bicarbonate is described as,

$$NH_4HCO_3 \triangleq NH_3\uparrow + H_2O + CO_2\uparrow \tag{3}$$

If other conditions remain unchanged, the forward reaction of ammonium bicarbonate can lead to more gas production, thereby increasing the system pressure. Assume that the amount of sodium bicarbonate is 1 mol. According to the chemical reaction equation, 2 mol of gas is produced. Under standard atmospheric pressure, T=333.15 K, the volume produced is calculated using the ideal gas equation,

$$V_{gas} = \frac{n_{gas}RT}{P} \tag{4}$$

The decomposition of ammonium bicarbonate can produce 0.05467 m^3 of gas. Based on the volumes of reactants and products, the net flow factor $f_{n,v}$ can be derived,

$$f_{n,v} = \frac{V_{\text{gas}}}{V_{NH_4HCO_3}} \tag{5}$$

The net flow factor for the thermal decomposition of ammonium bicarbonate is 1100.3. Based on the mass of reactants and the volume of products, the net flow rate $f_{n.m}$ can be derived,

$$f_{n,m} = \frac{V_{\text{gas}}}{m_{NH_4HCO_3}} \tag{6}$$

The net flow rate of thermal decomposition of ammonium bicarbonate is 0.692 m^3/kg. Considering that the efficiency of pressure generation cannot be 100%, the pressure volume work of the product should be lower than the energy of the reactants. Theoretically, the maximum pressure volume work is,

$$W_{1,2} = n \cdot R \cdot T \cdot \ln\left(\frac{P_2}{P_1}\right) = P_1 \cdot V_1 \cdot \ln\left(\frac{P_2}{P_1}\right) = P_2 \cdot V_2 \cdot \ln\left(\frac{P_2}{P_1}\right) \tag{7}$$

Assuming that the generated gas is released into an environment of 4 kPa (absolute pressure P_2 = 1.01325 × 105 Pa) at a relative pressure of 100 kPa (absolute pressure P_1 = 2.01325 × 105 Pa), the theoretical maximum pressure-volume work is -3.803 kJ. The effective specific energy of a closed system with solid reactants can be calculated as follows,

$$\omega_{eff,m} = \frac{-W_{1,2}}{m} \tag{8}$$

The effective specific energy of the system is 48.14 kJ/kg, and the effective net energy density can be calculated according to the following formula,

$$\omega_{eff,v} = \frac{-W_{1,2}}{V} = \frac{P_1 \cdot V_1 \cdot \ln\left(\frac{P_2}{P_1}\right)}{V} = -P_1 \cdot \ln\left(\frac{P_2}{P_1}\right) \tag{9}$$

The effective specific energy density is 77.084 MJ/m3.

8. Mathematics model of the "organ" growth

During the expansion of plant cells, their extension behavior can be described by a viscoplastic model that relates the pressure inside the cell to the linear extension rate of the cell. This model usually takes the following form of mathematical expression,

$$r = \varphi(P - Y)^n \tag{10}$$

Where Y is the yield pressure, and the cell will only extend when it exceeds this pressure, r is the current elongation rate, that is, the increase ratio of the cell length relative to the original length, P is the pressure inside the cell, φ is extensibility, that is, the increase ratio of the cell length relative to the original length, and n is a power term, usually close to 1, which represents the relationship between pressure and elongation.

$$PA = YA + (\frac{1}{\varphi}v)^{\frac{1}{n}}A \tag{11}$$

The area parameter A and the tip velocity parameter v are used to replace the value of the normalized velocity r.

9. Durability test

Ideally, the reversible chemical reaction takes place in a closed container and does not decay as the number of cycles increases. In practice, since it is impossible to achieve absolute sealing, there is a certain degree of leakage of the gas produced by the thermal decomposition of ammonium bicarbonate. After our multiple tests, 5-gram ammonium bicarbonate powder will lose an average of 0.07g after completing the growth of an "organ" (growth length, 15cm), retraction, and cooling, which is about 1.4% of the total weight. The leakage has little effect on the number of cycles. In addition, we can increase the number of cycles by supplementing raw materials.

10. Fixed-wing pluripotent drone

This twin-engine aircraft uses foam material as the fuselage structure (fig. S8), and the overall weight is as light as 51 g. Its wing design adopts the Clark Y-type airfoil, and the angle of attack of the wing is set to 6 degrees based on the simulation analysis results (Fig. 6c). To drive the drone, two 3.7-volt DC motors are equipped. In the pursuit of extreme lightweight, we select a 1.5-gram flight control system (AR3201, FlySky, China) that integrates a receiver and an electronic speed controller (ESC). Due to the high-speed flight characteristics of fixed-wing, changes in the "organ" will significantly affect the attitude of the drone. Therefore, we considered changing the "organ" in a stable state (e.g., static state) or making small changes to ensure the attitude of the drone. At the same time, we also took advantage of this. In the fixed-wing experiment, we used R-growth to change the lift coefficient of the drone, realizing the

wing growth and flight of the fixed-wing. Drone “organs” for wings are mainly used in micro drones. In actual use, facing higher wind resistance or external loads, we can add a more pressure-resistant restrictive layer (e.g., nylon cloth, silicone, etc.) to the outer layer of the "organ" and increase the internal pressure to achieve stronger rigidity to bear high wind resistance and external loads to cope with more severe environments.

11. Tri-rotor pluripotent drone

This tri-rotor pluripotent drone (fig. S10) has a wheelbase of 90 mm. Three tilting motors can provide vector power for the drone. This vector power and reprogramming growth enable the drone to achieve multi-environment capabilities while maintaining a stable attitude. All electronic components are coated with a layer of one-component silicone rubber material (K-704L, Kafuter, China) and sealed, as shown in fig. S11. In addition, the air-water test of the motor is shown in fig. S12. We choose a 915MHz receiver to meet the needs of underwater communication. Reprogramming growth is controlled by a LoRa32 control board (Heltech Automation, China). Since both multi-rotor and VTOL have the ability to hover stably and the "organ" is very light, changes in the shape, structure, or function of the "organ" will not affect the attitude of the drone.

12. Quadrotor pluripotent drone

The quadrotor pluripotent drone can expand its manipulation and sensing capabilities through R-growth. Specifically, it can grow grippers or sensors (fig. S14A). Tactile sensors have a built-in conductive layer, such as conductive thermoplastic polyurethane or liquid metal, and when they encounter a collision, their resistance changes trigger a signal. In some environments where vision and GPS are limited, tactile perception can be used as a way for drones to locate and navigate, similar to a human walking in the dark.

13. Detection through R-growth

It is dangerous and challenging for drones to detect an unknown object deep in a complex jungle. The pluripotent drone can grow a slender arm to pass the camera into the dense bushes. In the pluripotent drone system presented in Fig. 7b, a pair of visual sensors are integrated, and arranged at the front end of the drone body and the arm to be grown. The drone takes off and grows to conduct aerial reconnaissance of dense vegetation areas. Although the camera on the drone body can obtain an external view of the vegetation, it is difficult to identify objects inside the vegetation. In contrast, as the arm grows, the camera on the arm can penetrate deeper into the vegetation for more detailed detection. At T = 31.49 s, the arm penetrates the vegetation layer and successfully detects the tennis ball hidden in it. This process reflects the system's efficient detection performance in complex environments.

14. Perching through R-growth

The perching of the pluripotent drone relies on the principle of adsorption (Fig. 7d). Inside the "organ" is a silicone tube that provides negative pressure, one end of which is connected to a suction cup, and the other end is connected to a negative pressure pump. There is also a reinforcing rope, one end of which is connected to a suction cup, and the other end is connected to a motor for ascent. The drone takes off and grows an "organ" for adsorption. At T=14.19 s, the suction cup adheres to the ceiling (with a small hole) and rises along the reinforcing rope. At T=20.18 s, the drone touches the ceiling. The drone has the ability to perch far away from the contact surface. The drone can adhere to the ceiling with deep ravines through R-growth, which is similar to the rock surface of a cave. Unlike other perching drones, the pluripotent drone grows an adsorption "organ" through R-growth, which can perch far away from the contact surface.

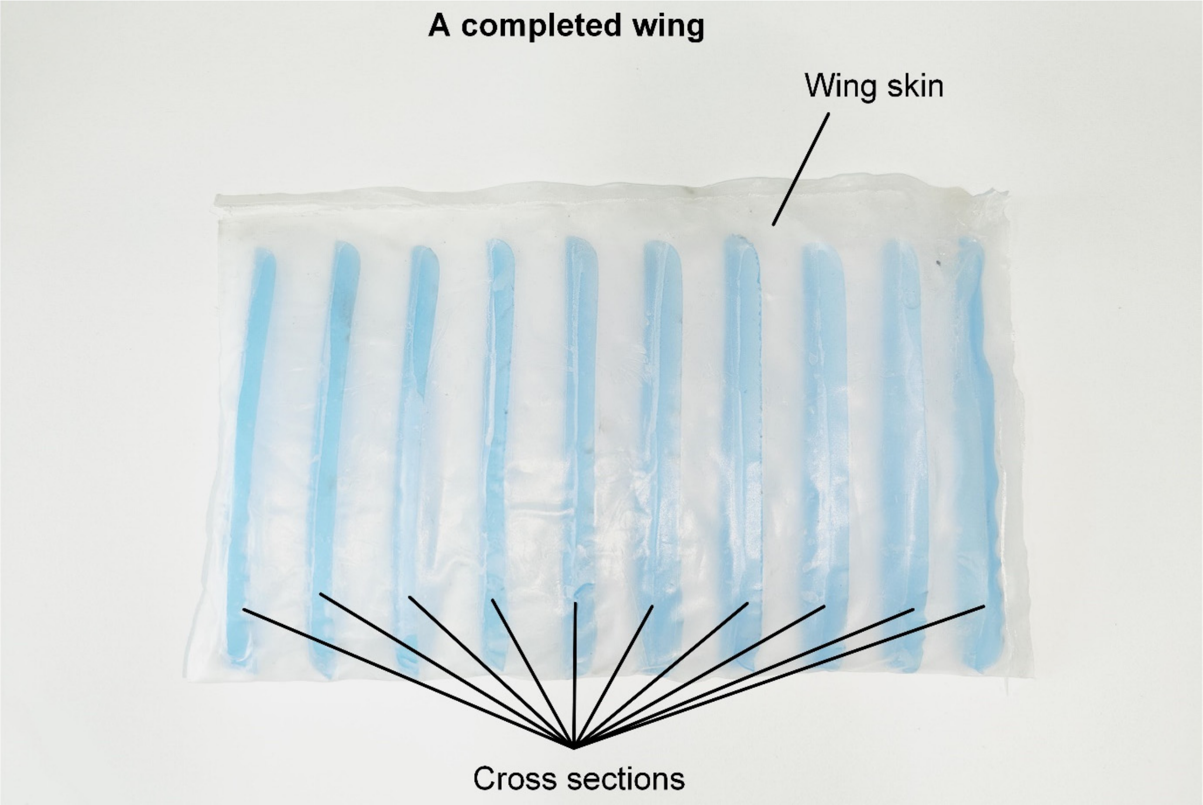

Fig. S1. A completed wing's image.

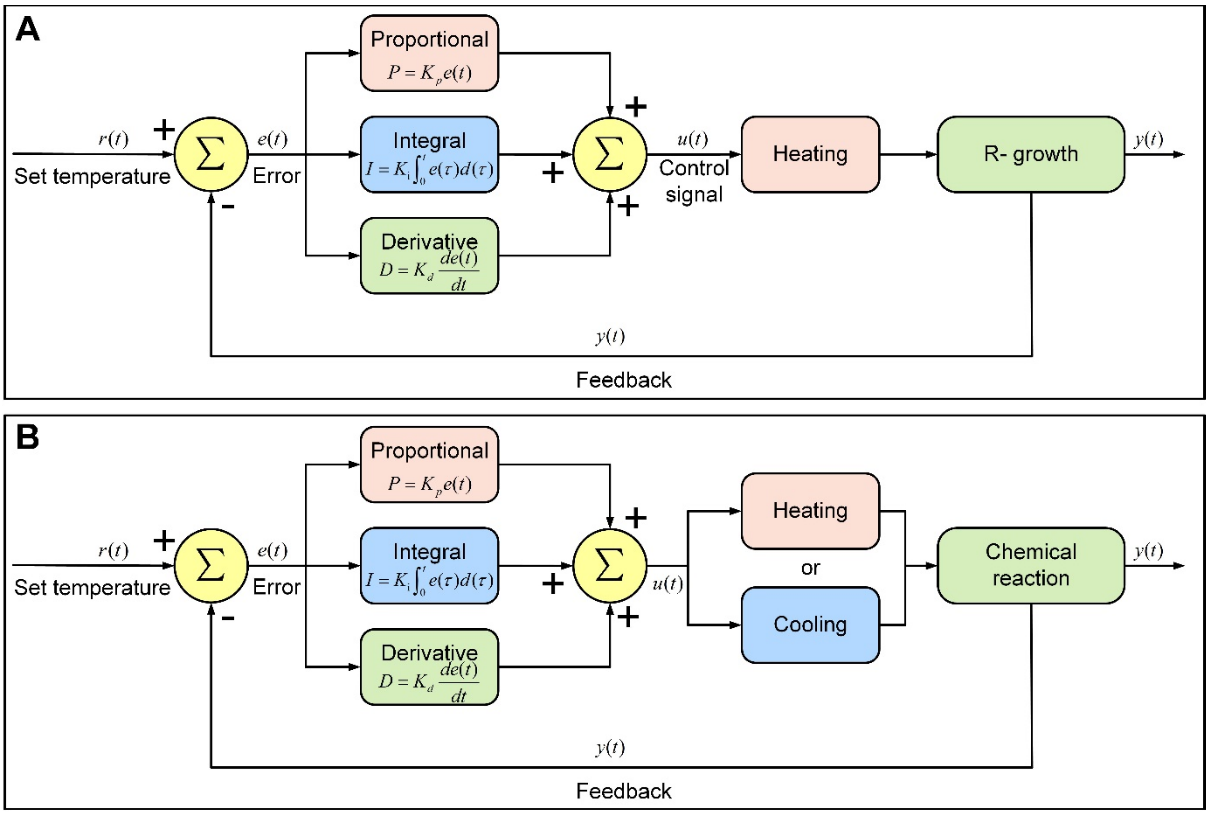

Fig. S2. Proportional-Integral-Derivative (PID) algorithm. (**A**) PID control block diagram for temperature field. (**B**) Block diagram of bidirectional PID control for thermal management of thermoelectric materials.

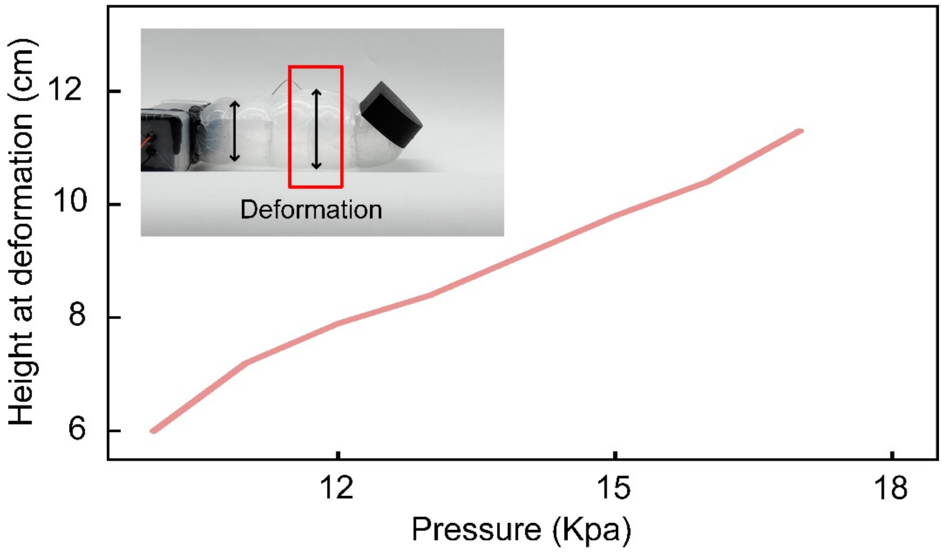

Fig. S3. Shape alteration in response to varying pressure through reprogramming growth.

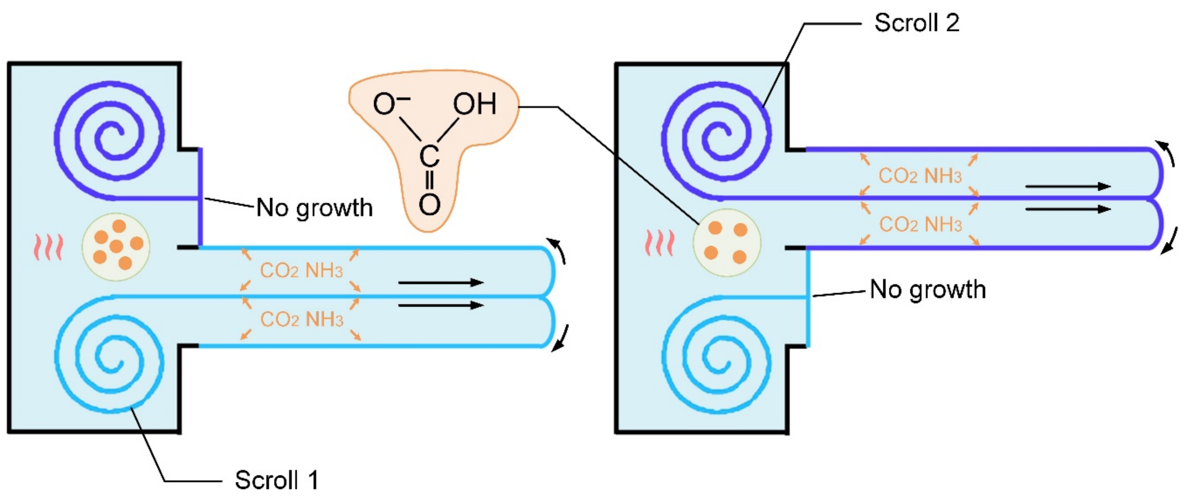

Fig. S4. Principles of reprogramming switching. The growth unit contains multiple scrolls that can grow into "organs", which are pre-programmed into different shapes.

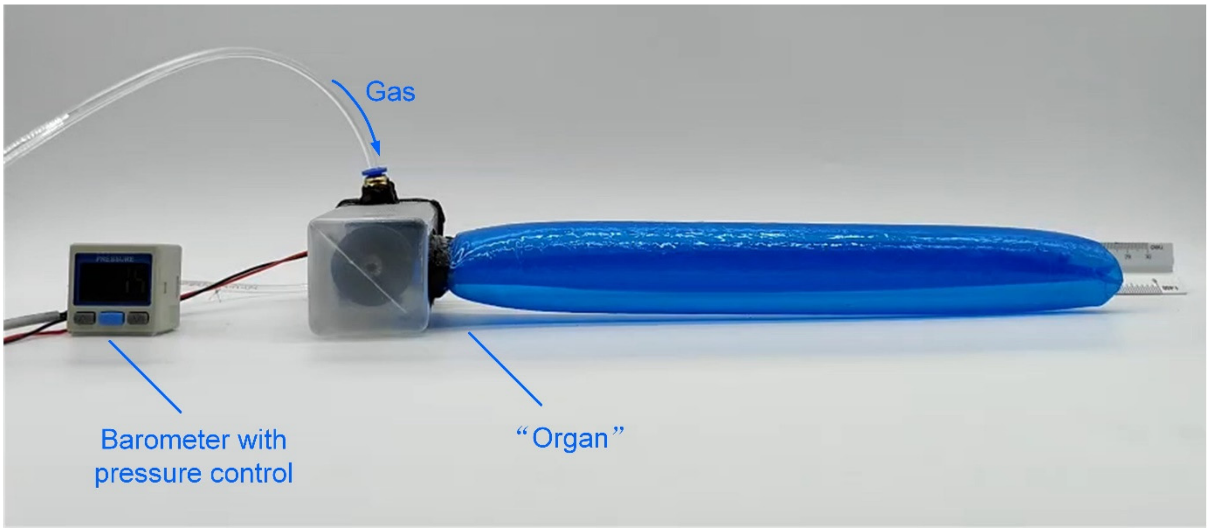

Fig. S5. Growth velocity tests under varying pressures.

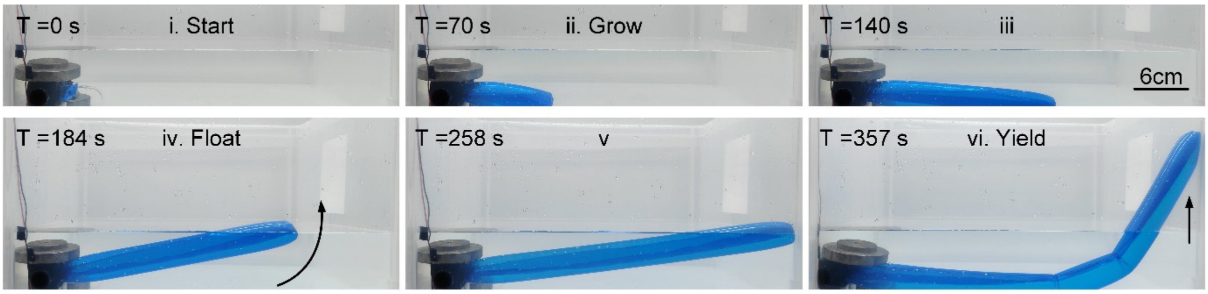

Fig. S6. Images of the "organ" growing underwater.

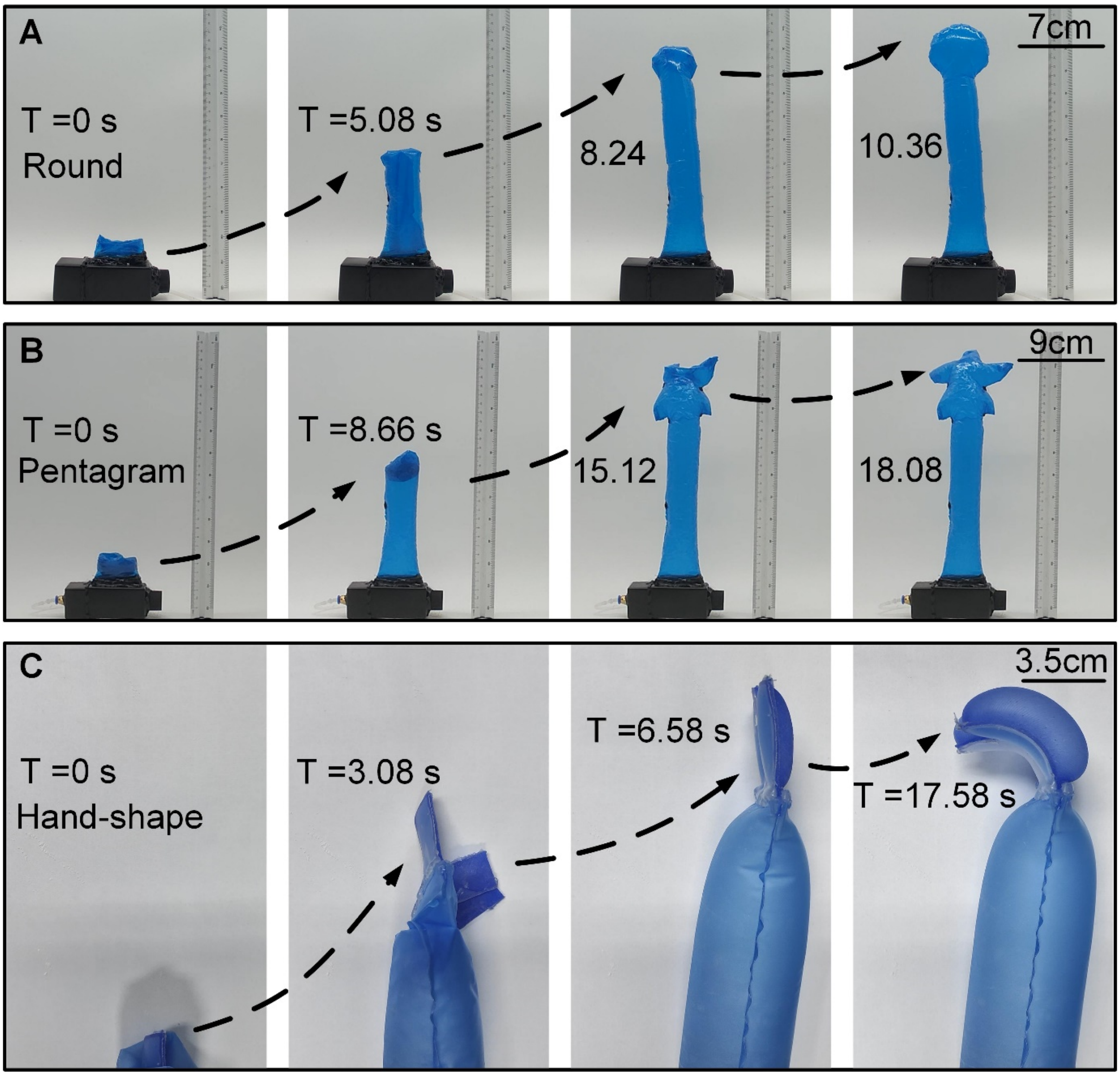

Fig. S7. Growth images of "organs" with pre-programmed shapes. (**A**) The growth of the round “organ”. (**B**) The growth of the pentagram “organ”. (**C**) The growth of the hand-shape “organ”. One side of the finger is made of silicone rubber. When the internal pressure increases, the difference in stretching rate enables the finger to bend and grasp.

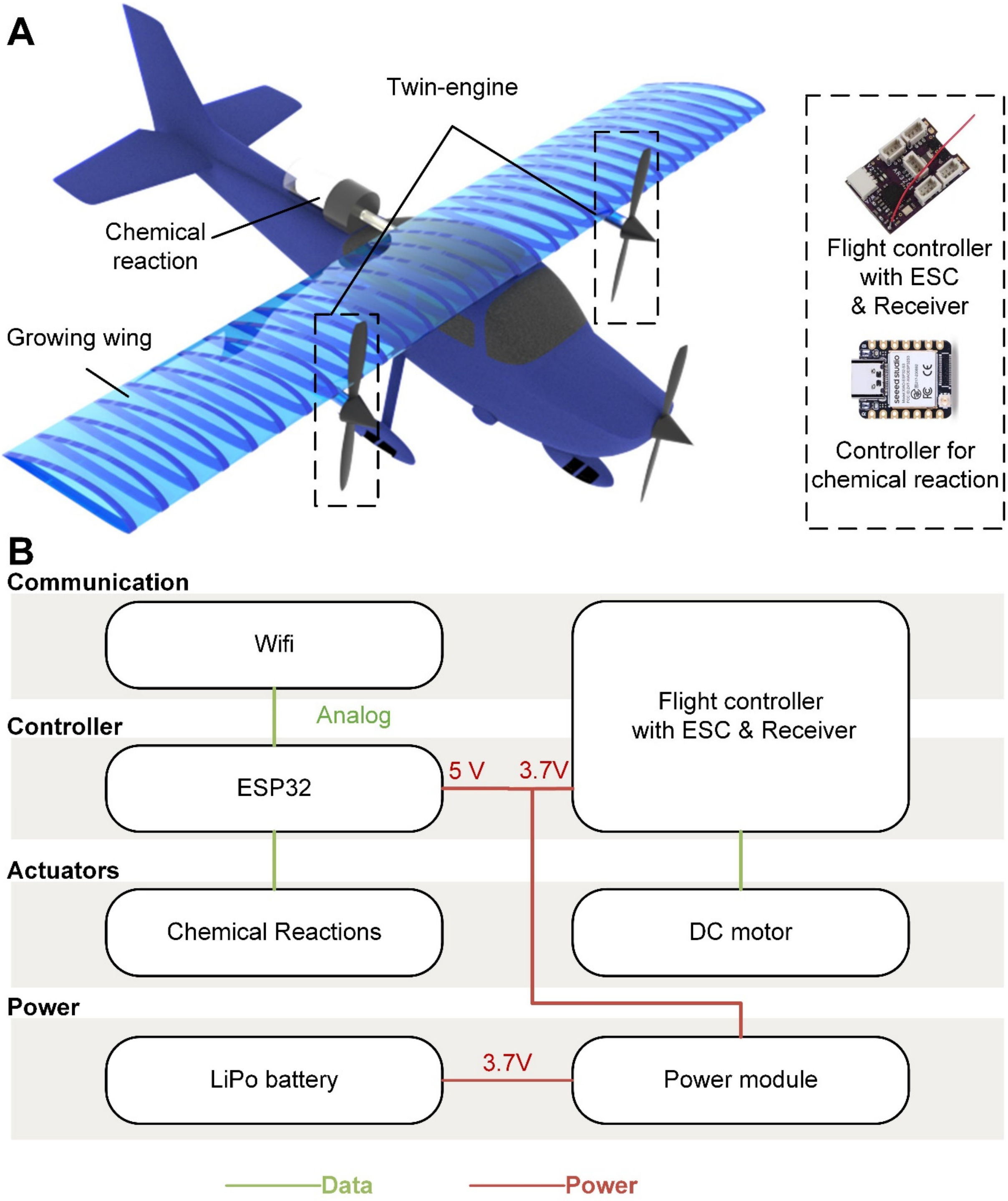

Fig. S8. Architecture of a lightweight pluripotent fixed-wing drone. (**A**) Configuration design for a fixed-wing airframe. Wing growth is driven by 5-gram chemical reaction (**B**) Electrical system of the fixed-wing drone.

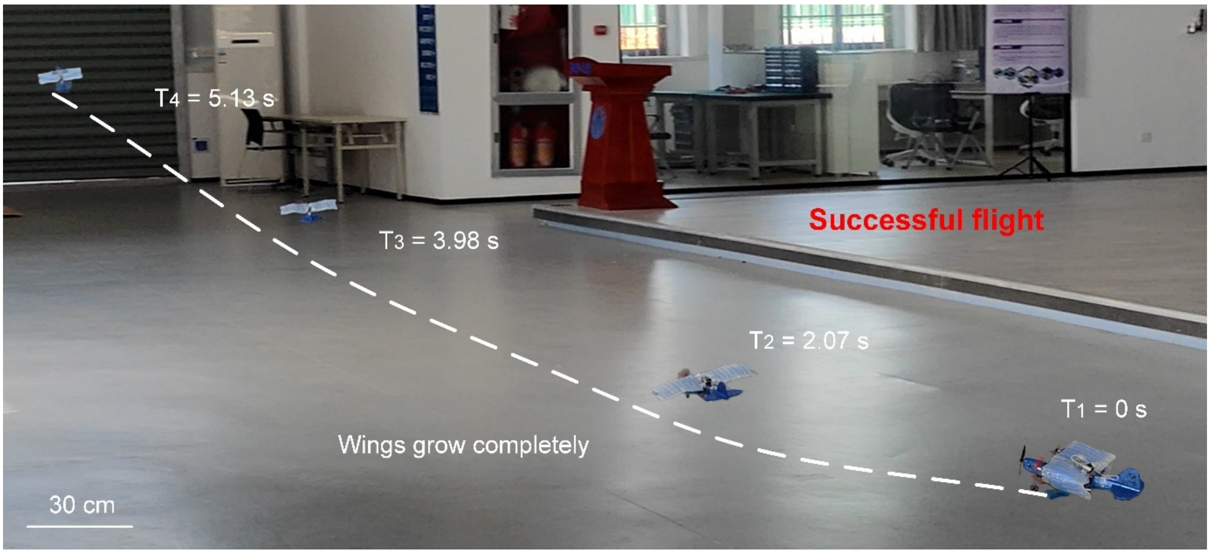

Fig. S9. Indoor testing scenario for the fixed-wing drone's performance.

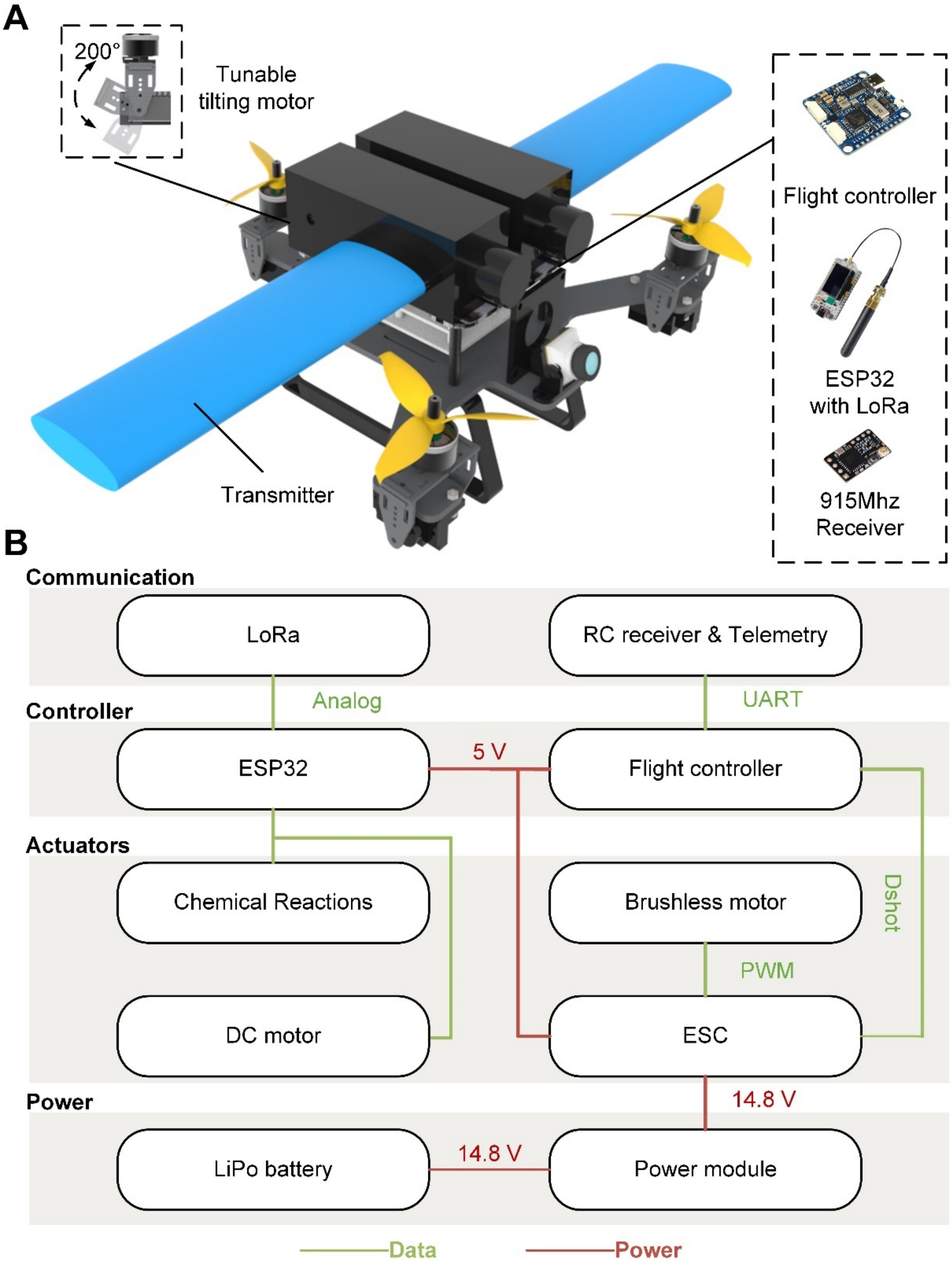

Fig. S10. Architecture of a tri-rotor drone. (**A**) Configuration design for a tri-rotor airframe. The tilting mechanism is capable of achieving an extreme tilt of up to 200 degrees. (**B**) Electrical system of the tri-rotor drone.

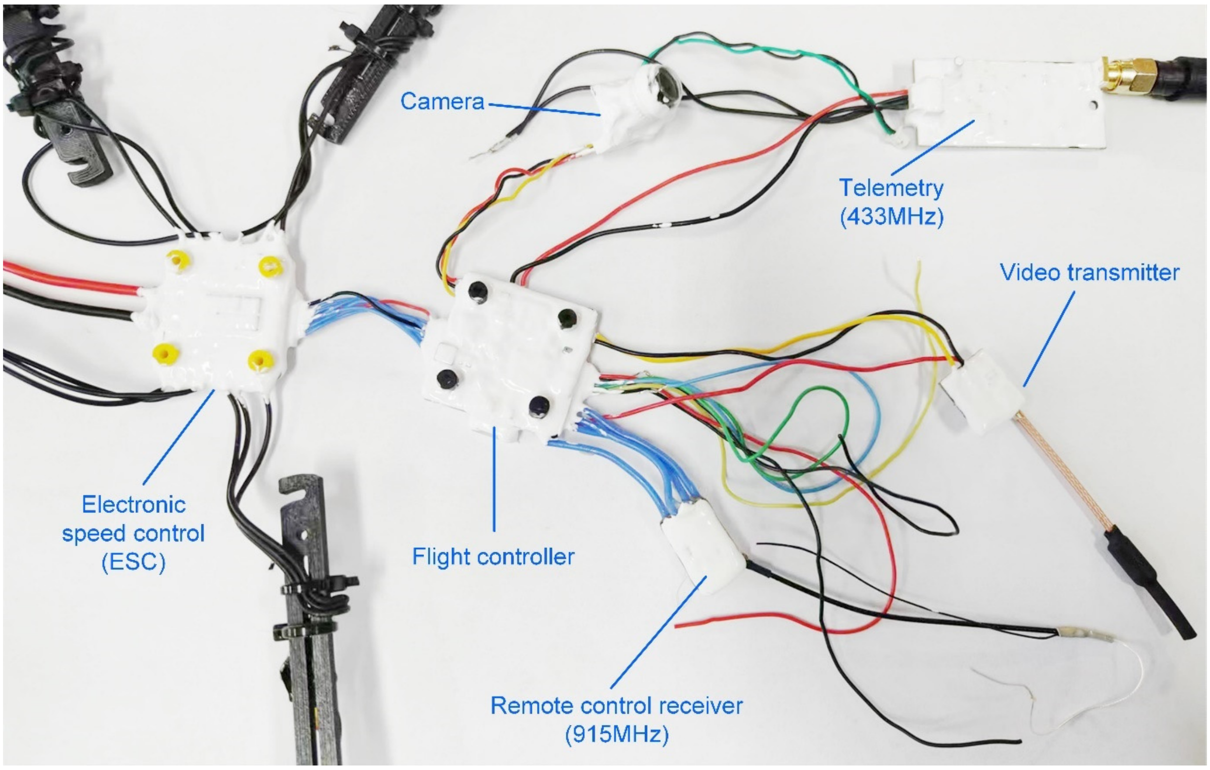

Fig. S11. Waterproof drone hardware.

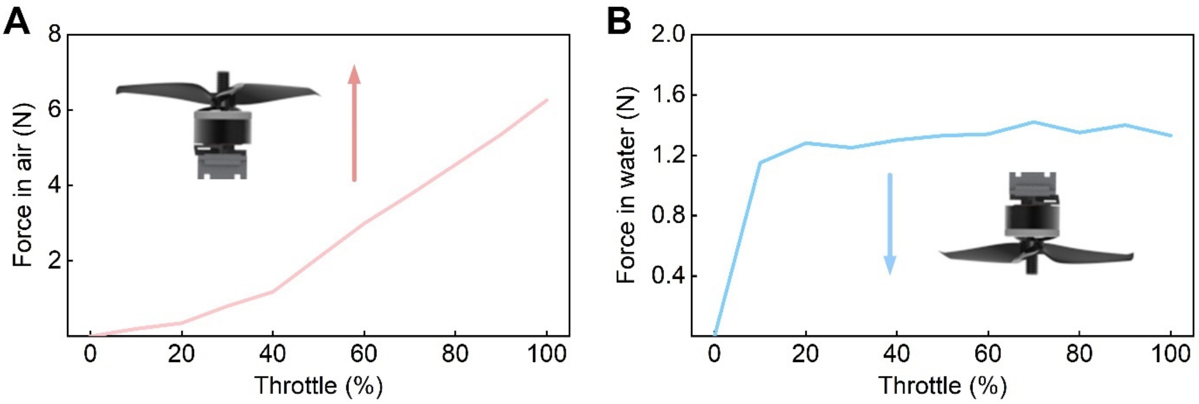

Fig. S12. Thrust measurement experiment for a brushless motor equipped with a 3-inch propeller. (**A**) Aerial thrust. (**B**) Underwater thrust**.**

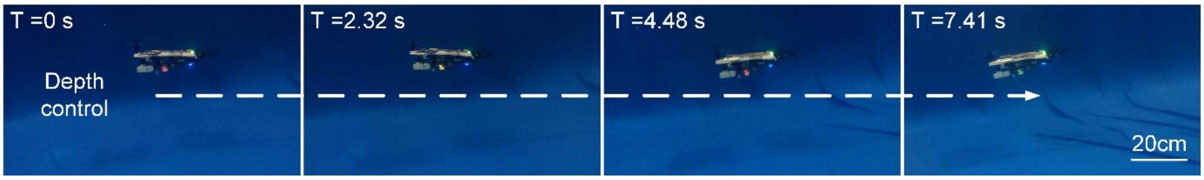

Fig. S13. Depth-fixed swimming images of the drone.

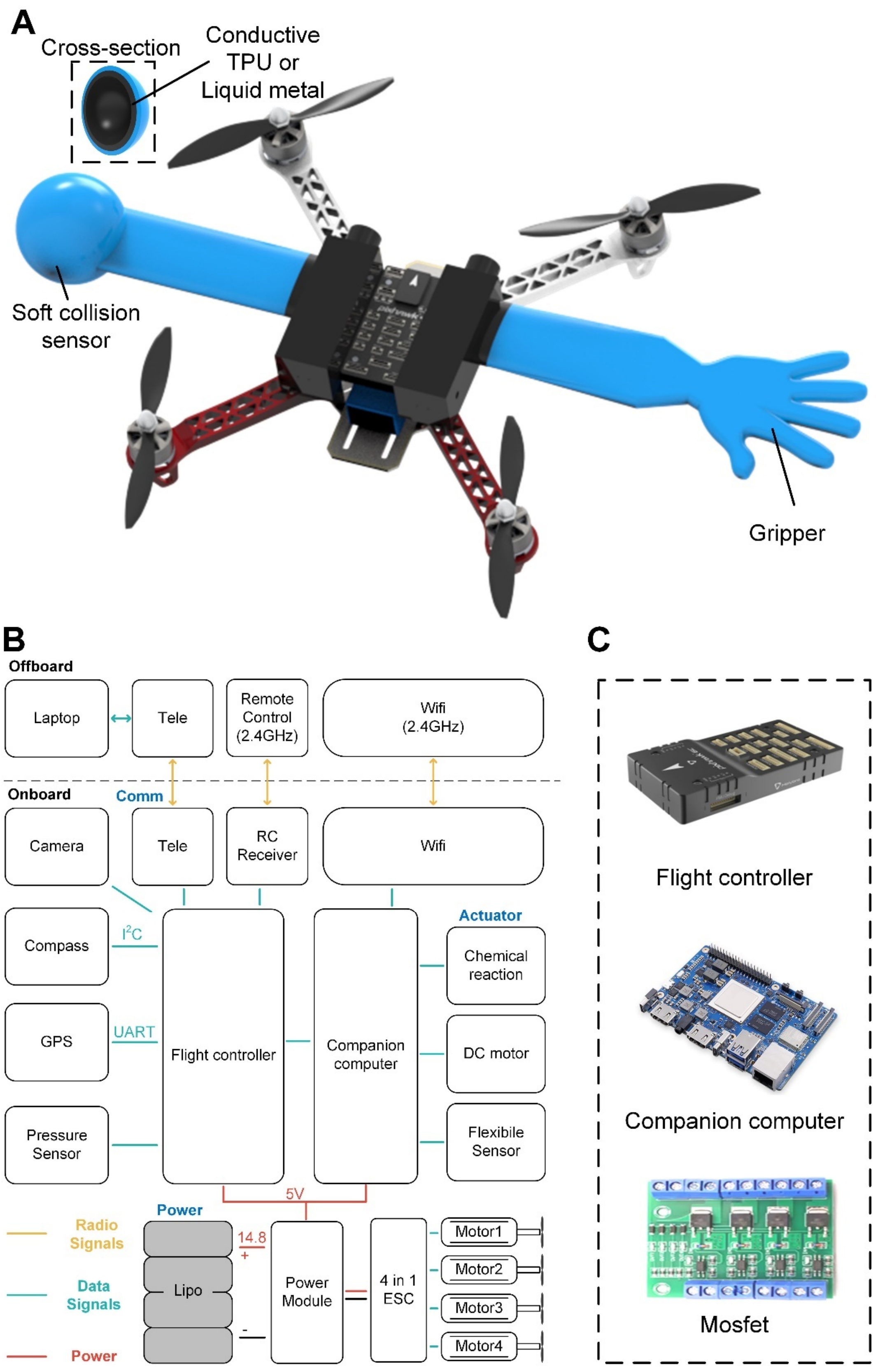

Fig. S14. Architecture of a quadrotor drone. (**A**) Configuration design for a quadrotor airframe, which is able to differentiates into grippers or touch sensors (**B**) Electrical system of the quadrotor drone.

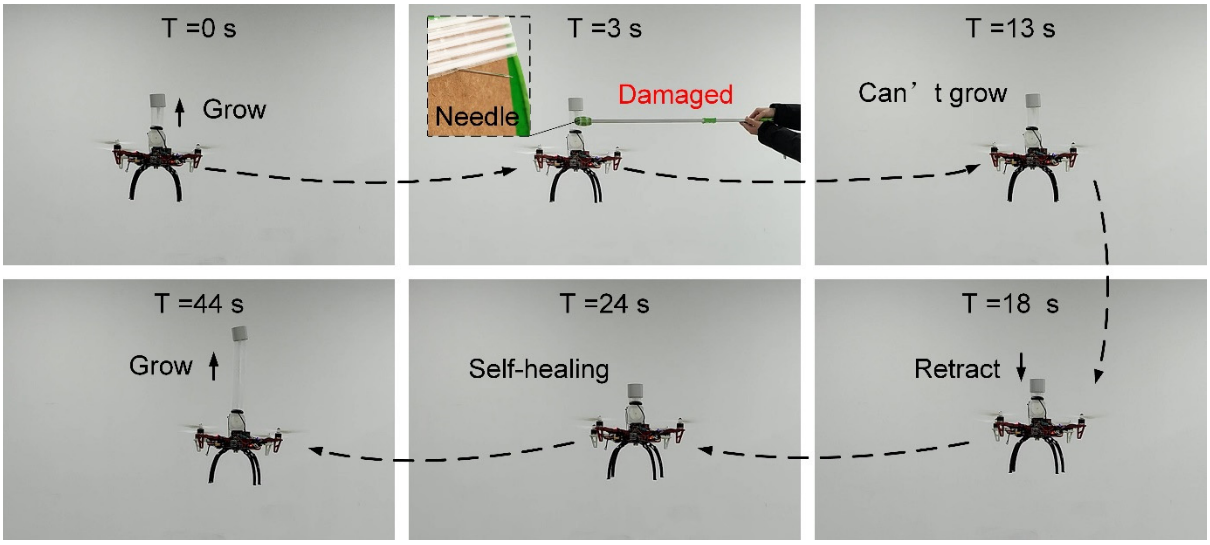

Fig. S15. “organ” self-healing of a quadrotor drone.

Table S1. Comparison with existing drones.

Drone	Functions of drones				
	Maneuver	Aerial-aquatic	Manipulation/ Grasping/Detection	Perching	Gap flight
Bioinspired drone actuated using wing [S1]	√				
Continuous morphing wing drone [S2]	√				
Bird-like morphing drone [S3]	√				
Bioinspired wing and tail morphing done [S4]	√				
3D-printed bird-like morphing drone [S5]	√				
Multimodal aerial–aquatic robot [S6]	√	√			
Waterproof aquatic drone [S7]		√			
TJ-FlyingFish [S8]		√			
Morphable aerial-aquatic drone [S9]		√			
Remora-inspired drone [S10]		√			
Vertical takeoff and landing drone [S11]		√			
Tilt-rotor drone [S12]	√				
Jump-gliding drone [S13]		√			
Swept wing drone [S14]		√			
Gliding aerial underwater drone [S15]		√			
Fixed-wing aerial-aquatic drone [S16]		√			
Cross-domain drone [S17]		√			
Fixed-wing aerial-underwater drone [S18]		√			
Drone with a foldable arm [S19]			√		
Aerial manipulation of articulated objects [S20]			√		
Drone with a parallel manipulator [S21]			√		
Drone with a lightweight dual arm system [S22]			√		
Additive manufacturing drone [S23]			√		
Drone with millimeter-level manipulator [S24]			√		
Drone with compliant arm [S25]			√		
Bird-inspired drone for dynamic grasping and perching [S26]			√	√	

Perching and resting drone [S27]				√	
Ornithopter drone [S28]				√	
Powerful drone manipulation [S29]			√		
Drone with a robot hand [S30]			√	√	
Drone with grasping mechanisms [S31]			√		
Dynamic grasping with a "soft" drone [S32]			√		
Drone with in-flight morphing aerial gripper [S33]			√		
LOCATOR drone [S34]				√	
Perching-and-takeoff drone [S35]				√	
Drone for autonomous perching and take-off [S36]				√	
Aerial–wall drone [S37]				√	
Drone with a novel passive mechanism [S38]				√	
Electroadhesive perching of drones [S39]				√	
Drone aggressively perching [S40]				√	
Drone with a mechanism for perching [S41]			√	√	
Multi-modal mobility drone [S42]			√		
Origami-inspired foldable drone [S43]					√
Soft-bodied drone [S44]				√	
The foldable drone [S45]					√
SQUEEZE drone [S46]					√
Metamorphic drone [S47]				√	
Ring-shaped drone [S48]			√		
Agile drone [S49]					√
**Our work**	√	√	√	√	√

Table S2. Comparison with existing TPU self-healing methods.

TPU self-healing	Self-healing mechanism	Self-healing temperature	Self-healing time
NIR induced self-healing [S50]	Diels-Alderc reaction	100℃	48h
Preparation and properties of self-healing [S51]	Diels-Alderc reaction	110℃	10h
Triple roles of thermoplastic polyurethane [S52]	Diels-Alderc reaction	130℃	48
Self-healing soft pneumatic robots [S53]	Diels-Alderc reaction	80℃	24h
Hydroxyl-terminated polybutadiene-based polyurethane [S54]	Boronic ester bonds	130℃	3h
Highly stretchable, self-Healable, and adhesive polyurethane [S55]	Boronic ester bonds	25℃	1h
A highly stretchable and self-healable [S56]	Hydrogen bonds	70℃	12h
Mechanically robust self-repairing [S57]	Hydrogen bonds	48℃	30h
Polyurethane networks based on disulfide bonds [S58]	Disulfide bonds	55℃	12h
Self-healing, reprocessing and sealing abilities [S59]	Disulfide bonds	75℃	48h
High performance and near body temperature [S60]	Multiple bonds	40℃	2h
Self-healing polyurethane [S61]	Multiple bonds	60℃	6h
**Our work**	Hot shrink	110℃	3.2s

Movie S1. Self-healing of a developed "organ".

Movie S2. Self-healing of a growing "organ".

Movie S3. Chemical growth mechanism.

Movie S4. Customizable preprogramming shapes of the "organ".

Movie S5. Reprogramming adjustment of the "organ".

Movie S6. Reprogramming switch of the "organ".

Movie S7. Pluripotent drone growing "organs" - wings.

Movie S8. Pluripotent drone retracting "organs" for gap flight.

Movie S9. Pluripotent drone growing "organs" for aerial-aquatic movement.

Movie S10. Pluripotent drone growing a large-scale "organ" for room detection.

Movie S11. Pluripotent drone growing an adsorptive "organ" for perching.

Movie S12. Pluripotent drone self-healingthe damaged "organ".

Supplementary references

S1. J. Zhang, Y. Liu, L. Gao, B. Liu, Y. Zhu, X. Zang, J. Zhao, H. Cai, Bioinspired Drone Actuated Using Wing and Aileron Motion for Extended Flight Capabilities. *IEEE Robotics and Automation Letters* **7**, 11197–11204 (2022).

S2. L. Y. Matloff, E. Chang, T. J. Feo, L. Jeffries, A. K. Stowers, C. Thomson, D. Lentink, How flight feathers stick together to form a continuous morphing wing. *Science* **367**, 293–297 (2020).

S3. M. Brody, D. Podell, F. Corte Garcia, E. Munoz, S. Massey, E. Minassian, N. Gharibi, D. Lyon, B. Sanchez, P. L. Bishay, "MataGull: A lightweight bio-inspired non-flapping bird-like morphing drone" in *2023 Regional Student Conferences* (American Institute of Aeronautics and Astronautics, University at Buffalo, Buffalo, New York, United States of America, 2023; https://arc.aiaa.org/doi/10.2514/6.2023-72218).

S4. E. Ajanic, M. Feroskhan, S. Mintchev, F. Noca, D. Floreano, Bioinspired wing and tail morphing extends drone flight capabilities. *Sci. Robot.* **5**, eabc2897 (2020).

S5. P. L. Bishay, M. Brody, D. Podell, F. Corte Garcia, E. Munoz, E. Minassian, K. Bradley, 3D-Printed Bio-Inspired Mechanisms for Bird-like Morphing Drones. *Applied Sciences* **13**, 11814 (2023).

S6. K. Qin, W. Tang, Y. Zhong, Y. Liu, H. Xu, P. Zhu, D. Yan, H. Yang, J. Zou, An Aerial–Aquatic Robot with Tunable Tilting Motors Capable of Multimode Motion. *Advanced Intelligent Systems* **n/a**, 2300193.

S7. Y. H. Tan, B. M. Chen, "A Lightweight Waterproof Casing for an Aquatic UAV using Rapid Prototyping" in *2020 International Conference on Unmanned Aircraft Systems (ICUAS)* (2020), pp. 1154–1161.

S8. X. Liu, M. Dou, D. Huang, S. Gao, R. Yan, B. Wang, J. Cui, Q. Ren, L. Dou, Z. Gao, J. Chen, B. M. Chen, "TJ-FlyingFish: Design and Implementation of an Aerial-Aquatic Quadrotor with Tiltable Propulsion Units" in *2023 IEEE International Conference on Robotics and Automation (ICRA)* (2023), pp. 7324–7330.

S9. Y. H. Tan, B. M. Chen, "Design of a Morphable Multirotor Aerial-Aquatic Vehicle" in *OCEANS 2019 MTS/IEEE SEATTLE* (2019), pp. 1–8.

S10. L. Li, W. Liu, B. Tian, P. Hu, W. Gao, Y. Liu, F. Yang, Y. Duo, H. Cai, Y. Zhang, Z. Zhang, Z. Li, L. Wen, An Aerial–Aquatic Hitchhiking Robot with Remora-Inspired Tactile Sensors and Thrust Vectoring Units. *Advanced Intelligent Systems* **n/a**, 2300381.

S11. R.-A. Peloquin, D. Thibault, A. L. Desbiens, Design of a Passive Vertical Takeoff and Landing Aquatic UAV. *IEEE Robot. Autom. Lett.* **2**, 381–388 (2017).

S12. M. Hassanalian, R. Salazar, A. Abdelkefi, Conceptual design and optimization of a tilt-rotor micro air vehicle. *Chinese Journal of Aeronautics* **32**, 369–381 (2019).

S13. R. Zufferey, A. O. Ancel, A. Farinha, R. Siddall, S. F. Armanini, M. Nasr, R. V. Brahmal, G. Kennedy, M. Kovac, Consecutive aquatic jump-gliding with water-reactive fuel. *Sci. Robot.* **4**, eaax7330 (2019).

S14. M. Harms, N. Kaufmann, F. M. Rockenbauer, N. Lawrance, T. Stastny, R. Y. Siegwart, "Differential Sweep Attitude Control for Swept Wing UAVs" in *2020 International Conference on Unmanned Aircraft Systems (ICUAS)* (IEEE, Athens, Greece, 2020; https://ieeexplore.ieee.org/document/9214033/), pp. 166–175.

S15. C. Lyu, D. Lu, C. Xiong, R. Hu, Y. Jin, J. Wang, Z. Zeng, L. Lian, Toward a gliding hybrid aerial underwater vehicle: Design, fabrication, and experiments. *Journal of Field Robotics*, rob.22063 (2022).

S16. J. Moore, A. Fein, W. Setzler, "Design and Analysis of a Fixed-Wing Unmanned Aerial-Aquatic Vehicle" in *2018 IEEE International Conference on Robotics and Automation (ICRA)* (2018), pp. 1236–1243.

S17. W. Weisler, W. Stewart, M. B. Anderson, K. J. Peters, A. Gopalarathnam, M. Bryant, Testing and Characterization of a Fixed Wing Cross-Domain Unmanned Vehicle Operating in Aerial and Underwater Environments. *IEEE J. Oceanic Eng.* **43**, 969–982 (2018).

S18. Z. Wei, Y. Teng, X. Meng, B. Yao, L. Lian, Lifting-principle-based design and implementation of fixed-wing unmanned aerial–underwater vehicle. *Journal of Field Robotics*, rob.22071 (2022).

S19. S.-J. Kim, D.-Y. Lee, G.-P. Jung, K.-J. Cho, An origami-inspired, self-locking robotic arm that can be folded flat. *Sci. Robot.* **3**, eaar2915 (2018).

S20. M. Brunner, G. Rizzi, M. Studiger, R. Siegwart, M. Tognon, A Planning-and-Control Framework for Aerial Manipulation of Articulated Objects. *IEEE Robot. Autom. Lett.* **7**, 10689–10696 (2022).

S21. B. Stephens, L. Orr, B. B. Kocer, H.-N. Nguyen, M. Kovac, An Aerial Parallel Manipulator With Shared Compliance. *IEEE Robotics and Automation Letters* **7**, 11902–11909 (2022).

S22. A. Suarez, A. E. Jimenez-Cano, V. M. Vega, G. Heredia, A. Rodriguez-Castaño, A. Ollero, Design of a lightweight dual arm system for aerial manipulation. *Mechatronics* **50**, 30–44 (2018).

S23. G. Hunt, F. Mitzalis, T. Alhinai, P. A. Hooper, M. Kovac, "3D printing with flying robots" in *2014 IEEE International Conference on Robotics and Automation (ICRA)* (IEEE, Hong Kong, China, 2014; http://ieeexplore.ieee.org/document/6907515/), pp. 4493–4499.

S24. M. Wang, Z. Chen, K. Guo, X. Yu, Y. Zhang, L. Guo, W. Wang, Millimeter-Level Pick and Peg-in-Hole Task Achieved by Aerial Manipulator. *IEEE Transactions on Robotics* **40**, 1242–1260 (2024).

S25. A. Suarez, M. Perez, G. Heredia, A. Ollero, Cartesian Aerial Manipulator with Compliant Arm. *Applied Sciences* **11**, 1001 (2021).

S26. W. R. T. Roderick, M. R. Cutkosky, D. Lentink, Bird-inspired dynamic grasping and perching in arboreal environments. *Science Robotics* **6**, eabj7562 (2021).

S27. K. Hang, X. Lyu, H. Song, J. A. Stork, A. M. Dollar, D. Kragic, F. Zhang, Perching and resting—A paradigm for UAV maneuvering with modularized landing gears. *Sci. Robot.* **4**, eaau6637 (2019).

S28. R. Zufferey, J. Tormo-Barbero, D. Feliu-Talegón, S. R. Nekoo, J. Á. Acosta, A. Ollero, How ornithopters can perch autonomously on a branch. *Nat Commun* **13**, 7713 (2022).

S29. X. Guo, W. Tang, K. Qin, Y. Zhong, H. Xu, Y. Qu, Z. Li, Q. Sheng, Y. Gao, H. Yang, J. Zou, Powerful UAV manipulation via bioinspired self-adaptive soft self-contained gripper. *Science Advances* **10**, eadn6642 (2024).

S30. A. McLaren, Z. Fitzgerald, G. Gao, M. Liarokapis, "A Passive Closing, Tendon Driven, Adaptive Robot Hand for Ultra-Fast, Aerial Grasping and Perching" in *2019 IEEE/RSJ International Conference on Intelligent Robots and Systems (IROS)* (2019; https://ieeexplore.ieee.org/abstract/document/8968076), pp. 5602–5607.

S31. L. Hingston, J. Mace, J. Buzzatto, M. Liarokapis, "Reconfigurable, Adaptive, Lightweight Grasping Mechanisms for Aerial Robotic Platforms" in *2020 IEEE International Symposium on Safety, Security, and Rescue Robotics (SSRR)* (IEEE, Abu Dhabi, United Arab Emirates, 2020; https://ieeexplore.ieee.org/document/9292581/), pp. 169–175.

S32. J. Fishman, S. Ubellacker, N. Hughes, L. Carlone, "Dynamic Grasping with a 'Soft' Drone: From Theory to Practice" in *2021 IEEE/RSJ International Conference on Intelligent Robots and Systems (IROS)* (2021; https://ieeexplore.ieee.org/document/9635927), pp. 4214–4221.

S33. N. Zhao, Y. Luo, G. Wang, Y. Shen, A deployable articulated mechanism enabled in-flight morphing aerial gripper. *Mechanism and Machine Theory* **167**, 104518 (2022).

S34. N. Iversen, O. B. Schofield, E. Ebeid, "LOCATOR - Lightweight and Low-Cost Autonomous Drone System for Overhead Cable Detection and Soft Grasping" in *2020 IEEE International Symposium on Safety, Security, and Rescue Robotics (SSRR)* (2020; https://ieeexplore.ieee.org/abstract/document/9292591), pp. 205–212.

S35. H. Liu, H. Tian, D. Wang, T. Yuan, J. Zhang, G. Liu, X. Li, X. Chen, C. Wang, S. Cai, J. Shao, Electrically active smart adhesive for a perching-and-takeoff robot. *Sci. Adv.* **9**, eadj3133 (2023).

S36. A. Kalantari, K. Mahajan, D. Ruffatto, M. Spenko, "Autonomous perching and take-off on vertical walls for a quadrotor micro air vehicle" in *2015 IEEE International Conference on Robotics and Automation (ICRA)* (2015; https://ieeexplore.ieee.org/document/7139846), pp. 4669–4674.

S37. Q. Li, H. Li, H. Shen, Y. Yu, H. He, X. Feng, Y. Sun, Z. Mao, G. Chen, Z. Tian, L. Shen, X. Zheng, A. Ji, An Aerial–Wall Robotic Insect That Can Land, Climb, and Take Off from Vertical Surfaces. *Research* **6**, 0144 (2023).

S38. H. Hsiao, F. Wu, J. Sun, J. Zhao, "A Novel Passive Mechanism for Flying Robots to Perch onto Surfaces" in *2022 International Conference on Robotics and Automation (ICRA)* (2022; https://ieeexplore.ieee.org/abstract/document/9811671), pp. 1183–1189.

S39. S. Park, D. S. Drew, S. Follmer, J. Rivas-Davila, Lightweight High Voltage Generator for Untethered Electroadhesive Perching of Micro Air Vehicles. *IEEE Robot. Autom. Lett.* **5**, 4485–4492 (2020).

S40. S. Liu, Z. Wang, X. Sheng, W. Dong, Hitchhiker: A Quadrotor Aggressively Perching on a Moving Inclined Surface Using Compliant Suction Cup Gripper. *IEEE Trans. Automat. Sci. Eng.*, 1–12 (2023).

S41. H. W. Wopereis, T. D. Van Der Molen, T. H. Post, S. Stramigioli, M. Fumagalli, "Mechanism for perching on smooth surfaces using aerial impacts" in *2016 IEEE International Symposium on Safety, Security, and Rescue Robotics (SSRR)* (IEEE, Lausanne, Switzerland, 2016; http://ieeexplore.ieee.org/document/7784292/), pp. 154–159.

S42. E. Sihite, A. Kalantari, R. Nemovi, A. Ramezani, M. Gharib, Multi-Modal Mobility Morphobot (M4) with appendage repurposing for locomotion plasticity enhancement. *Nat Commun* **14**, 3323 (2023).

S43. D. Yang, S. Mishra, D. M. Aukes, W. Zhang, "Design, Planning, and Control of an Origami-inspired Foldable Quadrotor" in *2019 American Control Conference (ACC)* (2019; https://ieeexplore.ieee.org/abstract/document/8814351), pp. 2551–2556.

S44. P. H. Nguyen, K. Patnaik, S. Mishra, P. Polygerinos, W. Zhang, A Soft-Bodied Aerial Robot for Collision Resilience and Contact-Reactive Perching. *Soft Robotics*, soro.2022.0010 (2023).

S45. D. Falanga, K. Kleber, S. Mintchev, D. Floreano, D. Scaramuzza, The Foldable Drone: A Morphing Quadrotor That Can Squeeze and Fly. *IEEE Robot. Autom. Lett.* **4**, 209–216 (2019).

S46. K. Patnaik, S. Mishra, S. M. R. Sorkhabadi, W. Zhang, "Design and Control of SQUEEZE: A Spring-augmented QUadrotor for intEractions with the Environment to squeeZE-and-fly" in *2020 IEEE/RSJ International Conference on Intelligent Robots and Systems (IROS)* (2020; https://ieeexplore.ieee.org/abstract/document/9341730), pp. 1364–1370.

S47. P. Zheng, F. Xiao, P. H. Nguyen, A. Farinha, M. Kovac, Metamorphic aerial robot capable of mid-air shape morphing for rapid perching. *Sci Rep* **13**, 1297 (2023).

S48. Y. Wu, F. Yang, Z. Wang, K. Wang, Y. Cao, C. Xu, F. Gao, Ring-Rotor: A Novel Retractable Ring-Shaped Quadrotor With Aerial Grasping and Transportation Capability. *IEEE Robotics and Automation Letters* **8**, 2126–2133 (2023).

S49. V. Riviere, A. Manecy, S. Viollet, Agile Robotic Fliers: A Morphing-Based Approach. *Soft Robotics* **5**, 541–553 (2018).

S50. C. Lin, D. Sheng, X. Liu, S. Xu, F. Ji, L. Dong, Y. Zhou, Y. Yang, NIR induced self-healing electrical conductivity polyurethane/graphene nanocomposites based on Diels−Alder reaction. *Polymer* **140**, 150–157 (2018).

S51. M. Li, H. Ding, X. Yang, L. Xu, J. Xia, S. Li, Preparation and Properties of Self-Healing Polyurethane Elastomer Derived from Tung-Oil-Based Polyphenol. *ACS Omega* **5**, 529–536 (2020).

S52. L. Feng, X. He, Y. Zhang, D. Qu, C. Chai, Triple Roles of Thermoplastic Polyurethane in Toughening, Accelerating and Enhancing Self-healing Performance of Thermo-reversible Epoxy Resins. *J Polym Environ* **29**, 829–836 (2021).

S53. S. Terryn, J. Brancart, D. Lefeber, G. Van Assche, B. Vanderborght, Self-healing soft pneumatic robots. *Sci. Robot.* **2**, eaan4268 (2017).

S54. J. Li, Z. Ning, W. Yang, B. Yang, Y. Zeng, Hydroxyl-Terminated Polybutadiene-Based Polyurethane with Self-Healing and Reprocessing Capabilities. *ACS Omega* **7**, 10156–10166 (2022).

S55. Y. Yang, F.-S. Du, Z.-C. Li, Highly Stretchable, Self-Healable, and Adhesive Polyurethane Elastomers Based on Boronic Ester Bonds. *ACS Appl. Polym. Mater.* **2**, 5630–5640 (2020).

S56. Y. Wang, S. Wang, X. Zhou, P. Chen, Y. Liu, L. Ding, C. Gao, A highly stretchable and self-healable hyperbranched polyurethane elastomer with excellent adhesion. *Reactive and Functional Polymers* **181**, 105443 (2022).

S57. Z. Shen, J. Chen, G. Li, G. Situ, X. Ma, Y. Sha, D. Zhao, Q. Gu, M. Zhang, Y. Luo, Z. Luo, Mechanically robust self-repairing polyurea elastomers: The roles of hard segment content and ordered/disordered hydrogen-bonding arrays. *European Polymer Journal* **181**, 111657 (2022).

S58. X.-Y. Deng, H. Xie, L. Du, C.-J. Fan, C.-Y. Cheng, K.-K. Yang, Y.-Z. Wang, Polyurethane networks based on disulfide bonds: from tunable multi-shape memory effects to simultaneous self-healing. *Sci. China Mater.* **62**, 437–447 (2019).

S59. W. Gao, M. Bie, Y. Quan, J. Zhu, W. Zhang, Self-healing, reprocessing and sealing abilities of polysulfide-based polyurethane. *Polymer* **151**, 27–33 (2018).

S60. H. Wu, X. Liu, D. Sheng, Y. Zhou, S. Xu, H. Xie, X. Tian, Y. Sun, B. Shi, Y. Yang, High performance and near body temperature induced self-healing thermoplastic polyurethane based on dynamic disulfide and hydrogen bonds. *Polymer* **214**, 123261 (2021).

S61. X. Jian, Y. Hu, W. Zhou, L. Xiao, Self-healing polyurethane based on disulfide bond and hydrogen bond. *Polymers for Advanced Technologies* **29**, 463–469 (2018).
